# Plant growth-promoting rhizobacteria: dual roles in enhancing nutrition and mitigating climate-related abiotic stresses

**DOI:** 10.3389/fmicb.2026.1800561

**Published:** 2026-08-04

**Authors:** Pan Qi, Liang Haoyue, Liquan Zhao, Muneer Ahmed Khoso, Sindho Wagan, Jiali Zhang, Qing Wang, Bohan Jiang, Fei Huang, Wang Yuxin, Jiayi Ma, Ifra Khan, Nazir Ahmed Brohi, Khuzin Dinislam, Shenglin Li

**Affiliations:** 1 School of Life Science and Agriculture Forestry, Qiqihar University, Qiqihar, Heilongjiang, China; 2 College of Life Sciences, Northeast Agricultural University, Harbin, China; 3 Department of Pharmacology, College of Pharmacy, Harbin Medical University, Harbin, China; 4 Laboratory of Pest Physiology, Biochemistry, and Molecular Toxicology, Department of Forest Protection, Northeast Forestry University, Harbin, China; 5 Institute of Microbiology, University of Sindh, Allama I.I, Kazi Campus, Jamshoro, Pakistan; 6 Bashkir State Medical University, Department of General Chemistry, Ufa, Republic of Bashkortostan, Russia

**Keywords:** abiotic stress, biotic stress, mechanisms, nutrients, PGPR, phytohormones, plant immunity

## Abstract

Climatic stresses impede plant growth and development, leading to significant reductions in crop yield and biomass production. These challenges are exacerbated by global population growth and increasing desertification, which threaten global food security. Although advanced agricultural technologies, including smart irrigation systems, optimized cropping calendars, and stress-tolerant cultivars, have been developed to mitigate these stressors, their large-scale adoption remains limited due to high costs, technical complexity, and infrastructural constraints. Therefore, sustainable, eco-friendly, and cost-effective strategies are urgently required to ensure adequate crop productivity for the growing global population. At this crucial juncture, there is a pressing need to transition toward sustainable agricultural practices that strengthen plant resilience through natural and biological mechanisms. Plant growth-promoting rhizobacteria (PGPR) represent a promising biological approach for enhancing crop productivity by improving nutrient availability and mitigating the adverse effects of climate-induced abiotic and biotic stresses. This review uniquely integrates the biochemical, physiological, and molecular mechanisms of PGPR in plant nutrition and stress mitigation while critically analyzing contradictory field results and highlighting newly characterized strains and sustainable tools for climate-resilient agriculture.

## Graphical abstract

**Figure:**
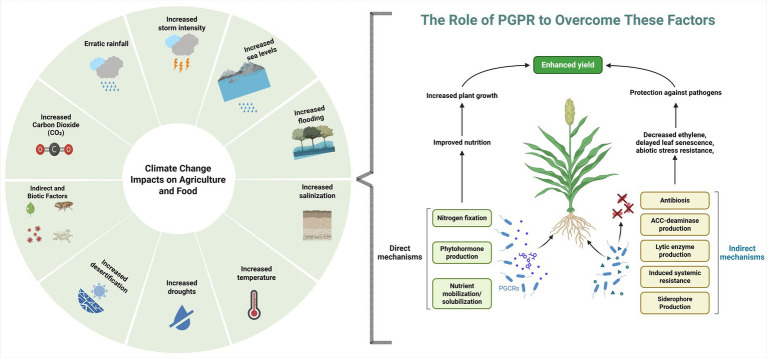

## 1 Introduction

Modern agriculture faces the pressing challenge of meeting the food demands of a rapidly growing global population without causing further environmental degradation. However, both the quality and quantity of important agricultural commodities, such as vegetables, pulses, and cereals, are affected by abiotic stresses, consequently undermining global food security (Haque et al., 2020; Jebara et al., 2021; Murthy et al., 2021). Abiotic stressors are estimated to account for 50–70% of worldwide agricultural output losses, especially during severe temperature events, drought, nutrient deficits, and salinity stress (Rahman et al., 2018; Abd El-Daim et al., 2019; Burdon and Zhan, 2020). Climate change severely affects numerous weather-dependent crops, including sorghum, maize, millet, barley, and rice, which collectively constitute major food sources worldwide (Ghadamgahi et al., 2022; Kumawat et al., 2022). In many practical applications, the development of management strategies for these stresses remains economically unfeasible or ineffective (Berni et al., 2022).

However, beyond nutritional enhancement, plant growth-promoting rhizobacteria (PGPR) regulate plant immunity by interacting with host plant signaling pathways and biotic stress factors (Kumari et al., 2024). PGPR naturally inhabit the rhizosphere and can gradually colonize root surfaces (rhizoplane) as epiphytes or internal root tissues (endophytes). Climate change is reducing crop yields and increasing agricultural costs, putting an additional 77 million people at risk of food insecurity by 2050. This is exacerbated by intensive agricultural practices, urbanization, and land-use changes. A significant increase in global temperature and the emergence of additional abiotic stressors adversely affect crop yield. In this context, eco-friendly technologies and biologically driven farming practices, including PGPR-based interventions, offer promising solutions for enhancing yields under increasingly environmentally challenging conditions and improving resource utilization.

Several reviews have addressed PGPR either for nutrient enhancement or stress mitigation separately, but a synthesis that incorporates both functions within the particular setting of climate-induced multiple stressors is absent. Therefore, the research gaps are as follows: (i) the lack of studies testing PGPR under combined abiotic stresses; (ii) the lack of strain-specific recommendations for various agroecological zones; and (iii) the inadequate understanding of why many PGPR strains fail to perform consistently from the laboratory to the field. This review provides a dual framework that links PGPR-mediated nutrient acquisition to stress tolerance pathways. It also critically evaluates contradictory results, highlights methodological flaws in current research, and focuses on newly characterized PGPR strains and their functional traits.

**Figure 1:**
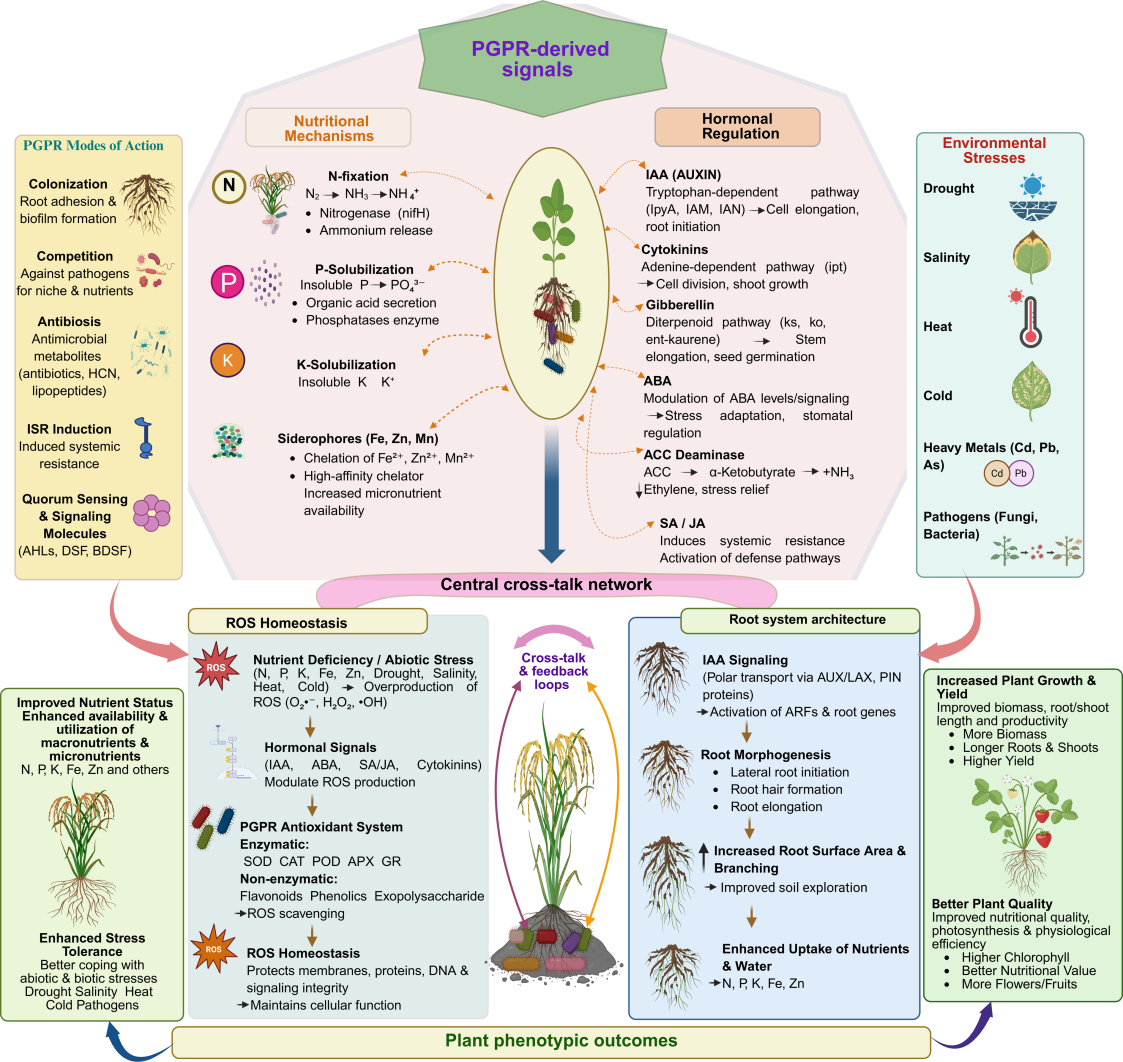
A graphical model depicting the interaction between PGPR-facilitated nutrient absorption mechanisms and hormonal regulatory pathways under abiotic stress. Created by the biorender.com.

Moreover, this study offers a detailed visual framework to elucidate the dual roles of PGPR, presenting a conceptual model (Figure 1) that clearly delineates the mechanistic interactions between nutrient absorption processes and phytohormonal regulatory mechanisms mediated by PGPR, including nutritional mechanisms such as nitrogen fixation, phosphorus solubilization, siderophore-mediated micronutrient acquisition, and potassium solubilization, as well as phytohormonal mechanisms involving indole acetic acid (IAA), ABA, cytokinins, gibberellins, SA/JA signaling, and ACC deaminase activity. The primary cross-talk network functions via two interconnected nodes: the homeostasis of reactive oxygen species (ROS), which links nutrient status and hormonal signals with antioxidant defense, and the modulation of root system architecture (RSA), which links IAA-mediated root growth to improved nutrient and water absorption, hence establishing a positive feedback loop. This comprehensive model emphasizes the synergistic processes that support climate-resilient agriculture and guides the ensuing discussion on PGPR-mediated stress reduction techniques. Furthermore, this study identifies priority areas for future research, including consortia engineering, native soil microbiome interactions, and genetic modification of PGPR for stress-specific traits.

## 2 The role of PGPR in climate change mitigation

Climate-induced stresses substantially reduce overall crop production; however, many current management strategies are costly, and certain stressors, specifically elevated temperatures, are difficult to mitigate under field conditions. Nonetheless, the application of PGPR has been shown to improve crop output by alleviating climate-related stressors via direct or indirect processes (Batool et al., 2020). PGPR directly enhance plant growth by modulating phytohormone levels, augmenting the availability of vital nutrients (e.g., nitrogen and phosphorus), and improving the bioavailability of iron through the release of siderophores (Kudoyarova et al., 2019; Wang et al., 2019). Beyond these direct benefits, PGPR also indirectly enhance plant health by inhibiting both established and emerging pathogens through antagonistic interactions, which include the synthesis of antibiotics, iron-chelating metabolites, and hydrolytic enzymes (Soenens and Imperial, 2020; Murthy et al., 2021). To enhance plant resilience against abiotic and biotic stresses associated with climate change, PGPR employ multiple, interlinked mechanisms, enabling plants to maintain growth and productivity under adverse conditions (Dhar Purkayastha et al., 2018; Bandyopadhyay et al., 2022).

Additionally, numerous studies have shown that inoculation with single PGPR strains or microbial consortia significantly enhances stress tolerance in cereal crops, such as maize, finger millet, rice, wheat, and sorghum. Global food security is also supported by horticultural crops such as potatoes and tomatoes, and current studies indicate that PGPR improve both stress resilience and productivity in these crops. However, PGPR-mediated benefits are highly strain-specific and influenced by the host plant, soil properties, environmental conditions, and genotype, highlighting the importance of targeted and crop-specific strain selection (Kelbessa et al., 2023). Furthermore, the following sections summarize the current knowledge of the molecular, biochemical, and physiological mechanisms by which PGPR mitigate plant responses to and adaptation to various climate-induced stresses.

## 3 Mechanistic role of PGPR in plant growth and development

The deficiency of nutrients in many agricultural regions worldwide, particularly in tropical areas where soil is severely depleted in nutrients, is the primary barrier to plant growth and development. Through several modes of action, PGPR contribute to biochemical nutrition cycles and regulate nutrient availability for plants and the soil microbial population. Using PGPR strains as bioinoculants can enhance soil nutrient availability, eliminate the need for synthetic fertilizers, reduce environmental pollution, and support sustainable agriculture. Numerous studies have investigated the improved health and production of various plant species following the application of PGPR under both normal and stressed conditions. These beneficial rhizobacteria reduce global reliance on harmful agrochemicals that disrupt agroecosystems. The predominant agricultural paradigm is excessively dependent on agrochemicals, leading to soil degradation and environmental contamination. It is important to promote environmental sustainability while ensuring food security; this may be accomplished by the application of PGPR, thereby reducing the need for synthetic agrochemicals. The direct mechanisms by which PGPR enhance plant growth include phytohormone synthesis, nitrogen fixation, nutrient solubilization, and the synthesis of siderophores and exopolysaccharides.

### 3.1 The role of PGPR in nutrient solubilization and uptake enhancement

The rhizosphere contains a diverse array of nutrient resources, and PGPR can enhance nutrient use efficiency and mobilize underutilized resources. The growth and development of plants depend on the availability of essential nutrients, and PGPR can facilitate nutrient acquisition by plants. They also influence many biogeochemical cycles involving nutrient components in the rhizosphere, thereby contributing to overall plant growth and development. To thrive, PGPR need to acquire various nutrients from the rhizosphere to support their multiplication, colonization, and competitive interactions, which explains why some groups, such as *Proteobacteria*, *Firmicutes*, and *Actinobacteria*, are among the most prevalent rhizosphere inhabitants. In the following section, we discuss the most recently characterized bacterial traits and their mechanisms for acquiring and utilizing these nutrients through diverse strategies.

Although phosphorus (P) solubilization by PGPR has been consistently shown *in vitro*, field data exhibit variability. For example, *Pseudomonas putida* MTCC 5279 demonstrated phosphorus solubilization of 42.5 μg/mL in controlled environments (Singh et al., 2024b), but its effectiveness in calcareous soils diminished by 60% owing to elevated pH buffering. These inconsistencies underscore a significant knowledge gap: the majority of research lacks long-term field validation under multiple interacting stresses. Moreover, most phosphate-solubilizing bacteria (PSB) screens are conducted using tricalcium phosphate, while ignoring other insoluble forms such as iron or aluminum phosphates, which predominate in several agricultural soils.

#### 3.1.1 Phosphorus solubilization

**Figure 2:**
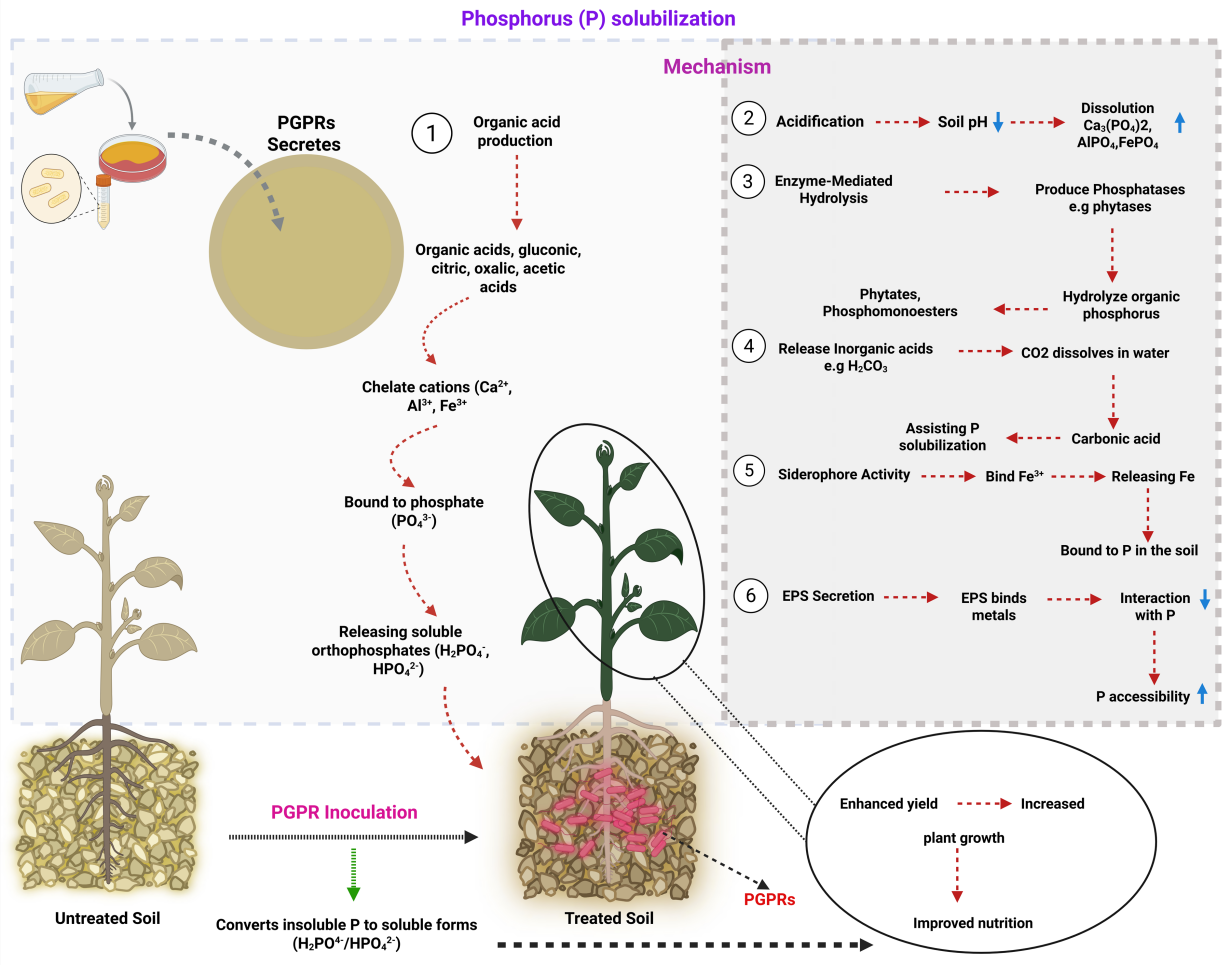
Overall mechanism of P solubilization by PGPR: PGPR bacteria solubilize phosphorus in soil by secreting organic acids and enzymes. These acids solubilize phosphate minerals locked in the soil, while the enzymes degrade organic phosphorus compounds. Ultimately, this process releases soluble phosphate for plant uptake, thereby stimulating growth and increasing yield. Created by the biorender.com.

Phosphorus is a primary macronutrient for plant growth, but it often exists in an insoluble form, rendering it unassimilable for plant uptake. In soil, PGPR solubilize complex insoluble phosphates, including aluminum phosphate, tricalcium phosphate, and rock phosphate, converting them into simpler soluble forms accessible to plants (Figure 2), thereby facilitating phosphate acquisition (Goswami et al., 2015; Goswami et al., 2016). Consequently, considerable research has focused on identifying effective PGPR strains that have the capacity to solubilize phosphorus (Table 1). These bacteria act synergistically to promote the solubilization of phosphorus and potassium, modify soil pH, improve soil structure, and facilitate the improvement of RSA in plants (Adnan et al., 2025).

**Table 1:** Examples of PGPR-mediated phosphorus (P) solubilization.

Plant	PGPR strain	Mechanism	Organic acids or enzymes	Tested insoluble P	Efficiency of P solubilization	References
*Zea mays*	*P. putida* MTCC 5279 (RA)	Secretion of gluconic acid and phosphatase activity	Gluconic and malic acids	Tricalcium phosphate (TCP)	42.5 μg/ml	Singh et al. (2024b)
Winter crops	*Enterobacter* sp. DRP3	Pyrroloquinoline quinone (PQQ)-dependent glucose dehydrogenase (gdh) gene	PQQ-dependent gdh gene and gluconic acid	TCP	655.89 μg/mL	Saha et al. (2024)
Maize	*Enterobacter cloacae* Rs-2	Secretion of organic acids and phosphatase	Gluconic acid and citric acid (genome-predicted)	Native soil P pool	Increased by 39.69 and 32.46%	Xue et al. (2024)
Rice	*Enterobacter asburiae* D2	Secretion of organic acids and phosphatase	Gluconic acid and citric acid (genome-predicted)	TCP	Increased by 57.1–150%	Ning et al. (2024)
Rice	*Pantoea vagans* ZHS-1	EMP–TCA cycle supplemented by other metabolic pathways	Pantothenic acid, maltose, glyoxylic acid, dicarboxylic acids	Sodium nitrate	Increased significantly	Tian et al., (2024)
	*Bacillus subtilis* ZE15	Increased phosphate esterase and exoenzyme activities	ß-D-glucosidase	Ca3(PO4)2	130 μg/mL	Iqbal et al., (2024)
Maize	*Bacillus anthracis* B1	Release of P from P-containing layered double hydroxide (LDH) compounds	Mg, Al, and LDH	Trisodium phosphate (TSP)	Increased significantly	Hassanzadeh et al. (2024)
Wheat	*Arthrobacter pascens*	Alleviation of drought and P deficiency	Palmitic, lactic, and stearic acids	NaCl	Increased by up to 68.91%	Benmrid et al. (2024)
Tomato	*Bacillus* BaT-68 s and *Pseudomonas* isolates	Induction of systemic immunity against the bacterial pathogen causing tomato canker	Rock phosphate (RP)	TCP	Up to 75% protection	Bakki et al. (2024)
Tomato	*Bacillus halotolerans* Z-13-4	P solubilization in liquid medium using several substrates (TCP, FeP, AlP, and bone meal)	Not mentioned	TCP, FeP, AlP	223.52 μg/mL; 140.3% increase	Haile et al. (2025)
Alfalfa	*Bacillus altitudinis AD13-4*	Affected signal transduction, photosynthesis, redox processes, primary and secondary metabolism, and plant–pathogen interactions	Not mentioned	Alkaline–sodic	Increased significantly	Khoso et al. (2024)

Furthermore, PSB are heterotrophic microorganisms that release low-molecular-weight organic acids to solubilize inorganic phosphate compounds. These acids increase the solubility of phosphate by acidifying the growth medium (Iftikhar et al., 2024).

Additionally, recent findings indicate that the examined bacterial strains belonging to PGPR genera, including *Kocuria*, *Bacillus*, *Pseudoxanthomonas*, *Agrobacterium*, *Priestia*, *Achromobacter*, and *Lysinibacillus*, identified via 16S rRNA gene sequencing, significantly contribute to phosphate solubilization (Rafique et al., 2025). In a study conducted in the tea-growing areas of the Kangra valley, a total of 493 rhizobacteria were recovered. Molecular fingerprinting of 160 morphotypes using ARDRA and ERIC analyses revealed 56 ERIC types and 52 rRNA types belonging to 21 distantly related genera. Subsequent 16S rRNA gene sequencing demonstrated both intergenic and intragenic variability. The molecular identification and diversity analysis of tea rhizobacteria from the Kangra valley, coupled with the recognition that phosphorus availability is a key growth-limiting factor for tea cultivation, suggested that the application of these effective PGPR strains may be beneficial for tea-growing soils. The most prevalent bacteria in the tea rhizosphere across different locations were *Bacillus altitudinis*, *Bacillus subtilis* subsp. *inaquosorum*, *Bacillus megaterium*, *Pseudarthrobacter mohnii*, *Pseudarthrobacter moriii*, and *Pseudarthrobacter frederiksbergensis*. Furthermore, 9 of 42 PGPR isolates demonstrated the ability to solubilize iron phosphate (Fe-P) and aluminum phosphate (Al-P) (Thakur et al., 2025).

Another study used 16S rRNA gene sequencing to identify several plant growth-promoting (PGP) traits, and the resulting genomes were compared with those in the NCBI database. The most effective PGPR inoculants based on the P-solubilization index were *P. nitroguahacolicus* and *V. paradoxus* in conventional and organic cropping systems (Flaviano et al., 2025). Additionally, another study on *Kabuli chickpea* evaluated five different phosphorus levels in combination with three biofertilizer treatments to identify the most effective inoculants. The combined application of *Mesorhizobium + PSB-18* and *Mesorhizobium + RB-1*, together with increasing phosphorus doses, significantly improved nutrient content and uptake in *Kabuli chickpea* (Kaur and Singh, 2025).

#### 3.1.2 Other important macronutrients

**Figure 3:**
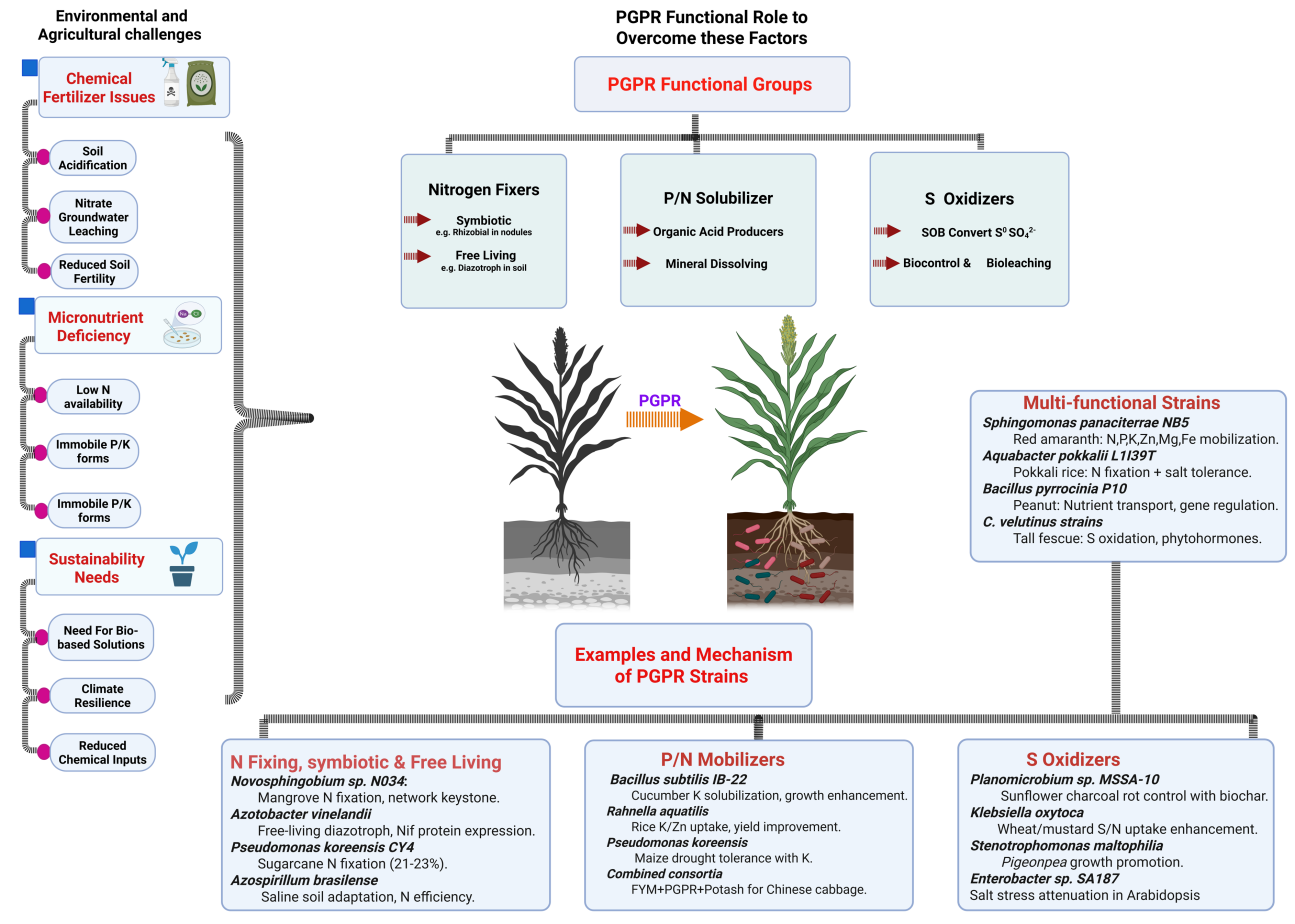
Mechanisms by which PGPR regulate macronutrient availability in plants: PGPR strains promote plant growth by fixing nitrogen, mobilizing essential nutrients, and solubilizing phosphorus and minerals. They enhance soil quality and enable plants to resist environmental stresses, such as drought and salinity. These functions lead to increased plant yield and nutrient uptake. Created by the biorender.com.

Agriculturally beneficial microbes can enhance crop growth and productivity through direct and indirect mechanisms, even though macronutrients such as N, K, and S supplied by mineral fertilizers are crucial for crop productivity (Figure 3). To counteract the short- and long-term adverse effects of low soil fertility, soil acidification, and nitrate leaching into groundwater, it is imperative that innovative, sustainable, and maintenance practices be adopted instead of chemical fertilizers. Therefore, efficient biological methods, such as the use of PGPB, have been increasingly explored to improve crop productivity (Dinesh et al., 2013).

##### 3.1.2.1 Nitrogen fixation

Nitrogen levels in agricultural soil are often low due to constant losses through leaching, volatilization, and denitrification; moreover, atmospheric nitrogen cannot be directly used by plants (Abd El-Aal et al., 2021). Therefore, biological nitrogen fixation and nutrient supplementation rely on rhizobacteria that promote plant growth and development. These nitrogen-fixing bacteria can be broadly classified as symbiotic, free-living, and associative diazotrophs (Raymond et al., 2004). Among these nitrogen-fixing bacteria, diazotrophs are particularly important for sustainable agriculture, as they simultaneously supply nitrogen to host plants and contribute to the suppression of plant-parasitic nematodes (Liao et al., 2021). Recent studies have shown that the co-culture of the free-living diazotroph *Azotobacter vinelandii* and the non-nitrogen-fixing PGPR *Bacillus subtilis* results in interactions that are strongly influenced by nitrogen availability. Under nitrogen-rich conditions, the growth of *B. subtilis* is enhanced, likely due to nitrogen transfer and competitive nutrient utilization. However, during the stationary growth phase, co-culturing *B. subtilis* and *A. vinelandii* enhanced nitrogen metabolism, as evidenced by increased levels of the molybdenum nitrogenase isoform proteins NifK and NifD in a proteomic study (Leroux et al., 2024). In sugarcane, nitrogen fixation was significantly enhanced by *Pseudomonas koreensis* CY4 and *Pseudomonas entomophila* CN11. The study demonstrated that these strains contributed to biological nitrogen fixation and have the potential to be used as biofertilizers to promote sugarcane growth, enhance nitrogen uptake, and reduce the use of synthetic nitrogen fertilizers in agricultural systems (Singh et al., 2024a). Another study employed a network-directed isolation technique to identify the keystone diazotrophic PGPR *Novosphingobium* sp. N034, which was able to optimize the composition of the sediment microbial community to support the growth of the mangrove plant *Kandelia obovata* (Huang et al., 2024).

##### 3.1.2.2 Potassium

Potassium is recognized as a highly dynamic macronutrient that significantly enhances root development, regulates osmotic balance, maintains ionic homeostasis, and promotes water retention, hence decreasing wilting and energy depletion (Polara et al., 2009). Alternative sources of K, including farmyard manure (FYM), potassium-solubilizing PGPR, and nano-potash, offer viable and sustainable options for potassium supplementation. FYM improves soil organic matter content when applied in conjunction with organic amendments, while nano-potash improves growth indices, yield, and quality across several crops (Afify et al., 2019). Recent studies have shown that the potassium application using muriate of potash (MOP; KCl), when integrated with FYM, shows promising results for improving yield and quality traits in Chinese cabbage (*Brassica rapa* L. subsp. *chinensis*) (Choudhary et al., 2024).

Additionally, recent studies have explored the role of *B. subtilis* in regulating cucumber seedling growth and the photosynthetic system under diverse K levels. In these studies, PGPR were used to solubilize soil potassium, providing theoretical support for further validating the effect of biofertilizers on cucumber growth and K uptake. Using the cucumber cultivar Xinjin No. 4 as the experimental material, a two-factor experimental design was employed to evaluate the effects of different treatments on seedling growth, root morphology, photosynthetic traits, and chlorophyll fluorescence parameters. The combined application of K and *B. subtilis* substantially enhanced cucumber seedling growth, photosynthesis, and leaf growth (Li et al., 2024). A recent study explored the role of the PGPR strains *Rahnella aquatilis* and *Burkholderia cepacia* in enhancing K and Zn uptake in rice (Bakhshandeh et al., 2024). Moreover, other PGPR strains, *P. koreensis* and *P. vancouverensis*, were investigated for their ability to enhance maize production and physiological traits under drought stress conditions (Samadi et al., 2024).

Potassium is the third-most abundant essential element in plants, accounting for 0.5–6% of their dry mass. It is involved in ATP synthesis, protein synthesis, photosynthesis, and the translocation of photosynthates (Hasanuzzaman et al., 2018). Additionally, potassium application combined with FYM and S effectively controls charcoal rot disease (Morsy, 2012). The effects of S application (0 to 2.25 mg/kg) in combination with FYM (2%), nitrogen, phosphorus, and potassium (NPK) (20:20:20 mg/kg), and three distinct PGPR strains were investigated to evaluate the suppression of charcoal rot disease and improvements in the growth yield, biochemical characteristics, and physiology of sunflower. The combined application of *Planomicrobium* sp. (strain MSSA-10), farmyard waste biochar, and 2.25 mg/kg S was effective in controlling charcoal rot disease and improving sunflower productivity (Ijaz et al., 2021b). Sulfur-oxidizing bacteria (SOB) convert elemental S into plant-accessible sulfate, which promotes plant development and enables the bacteria to function as biocontrol and bioleaching agents. Seed priming with SOB strains isolated from the rhizospheres of *Catharanthus*, *Trigonella*, *Spinacia*, *Begonia,* and *Azadirachta* improved seed vigor. All six SOB strains demonstrated *in vitro* PGP properties, making them promising candidates for sustainable agriculture and potential alternatives for organic fertilizers (Jadhav et al., 2024).

Another study identified the SOB isolates as *Klebsiella oxytoca*, *B. cepacia*, and *Enterobacter cloacae*, which enhanced S and N uptake and demonstrated their potential as PGPR with biofertilization capabilities in the mustard and wheat crops (Chaudhary et al., 2022). Similarly, *Pigeopea* growth and development were enhanced via two SOB strains isolated from coal mines, namely *Stenotrophomonas maltophilia* DRC-18-7A and *Stenotrophomonas pavanii* DRC-18-7B (Malviya et al., 2022). To overcome salt stress in *Arabidopsis*, the *Enterobacter* sp. SA187 attenuates the sulfur-starvation response induced by salt stress. Most sulfur metabolism takes place in chloroplasts and is associated with the accumulation of ROS. In SA187-colonized plants grown under either 0 or 100 mM NaCl, ROS accumulation was reduced, whereas non-colonized *Arabidopsis* exhibited increased ROS accumulation under salt stress. Inoculation with SA187 alleviated salt stress by modulating sulfur metabolism, which is closely linked to ROS detoxification and enhanced redox capacity, as reflected by an increased reduced-to-oxidized glutathione (GSH/GSSG) ratio, thereby strengthening sulfur metabolic pathways and stress tolerance in *Arabidopsis* (Mahmood et al., 2019b; Andrés-Barrao et al., 2021). Moreover, PGPR contribute to improving plant mineral nutrition by facilitating the utilization of key macronutrients such as N, K, and S (Table 2). These microbial functions support sustainable agricultural production by improving plant mineral nutrition and highlight the potential of microbiome-engineering approaches as powerful tools for enhancing plant growth, development, and resilience under changing environmental conditions.

**Table 2:** Role of PGPR in plant growth via macronutrients.

Plant	PGPR strain	Mechanism	Effect	Additional observations	References
Wheat	*P. mandelii* (IB-Ki14) and *B. subtilis* (IB-22)	Auxin enhances lignin and suberin production, cytokinin production, and hydraulic conductivity	Hormone-mediated, apoplastic control, lignification, and Casparian band formation	Improved shoot biomass, leaf area, chlorophyll content, and hydraulic conductivity	Akhtyamova et al. (2023)
Maize	*A. nigricans* (R19)	N fixation, P solubilization, and stress tolerance	Improved overall plant growth and development	Reduced NPK dependency and improved tolerance to abiotic stress	Sagar et al. (2022)
Mangrove	*Novosphingobium* sp. (N034)	N fixation, promotion of N retention, iron-reducing bacteria (FeRB), nitrate reduction, and suppression of anammox genes	Improved plant height, sediment NH4 + -N content, N content, and interactions with microbial the network	Alternative nitrate signaling pathway	Huang et al. (2024)
Tall fescue	*C. velutinus* (CK-06, 22, 44, 50)	Phytohormone production, nutrient solubilization, and sulfur oxidation	Improved fresh and dry weight	Sulfur-oxidizing properties	Devkota et al. (2024)
Red amaranth	*Sphingomonas panaciterrae* (NB5)	Nutrient solubilization, phytohormone production, and improvement of soil health	Increased plant growth (height, leaf number, root growth, stem growth, and chlorophyll content), antioxidant activity, and nutrient contents (N, P, K, Zn, Mg, and Fe) in shoots and roots; enhanced soil N, P, Ca, SOC, and S	Enhanced fibrous root development (RD, FA, and BCF)	Sultana et al. (2024a)
Pokkali rice	*Aquabacter pokkalii* (L1I39T)	N fixation, ACC deaminase activity, salt tolerance, and root colonization	Enhanced soil N content and improved saline conditions	Promoted salt adaptation and plant association; stimulated multiple genes	Sunithakumari et al. (2024)
Barley	*Parthenium hysterophorus and Serratia odorifera*	Enhanced antioxidant activity, improved drought tolerance, and enhanced nutrient uptake	Improved shoot length, fresh biomass, germination, dry mass, chlorophyll (a and b), and soil health (via increased K, P, and N contents and reduced electrolyte leakage)	ROS detoxification	Gul et al. (2023)
Peanut	*B. pyrrocinia* (P10)	Enhanced the transport of nutrients (carbohydrates, sulfur, nitrogen, and amino acids); upregulated genes involved in biofilm formation, quorum sensing, and secretion systems (types II, III, and VI); and activated genes involved in P solubilization, siderophore biosynthesis, and IAA production	Enhanced plant growth, rhizosphere colonization, and competitiveness against other microbes	Biofilm formation induced by citrate, malic acid, and oxalic acid	Han et al. (2023)
*Momordica charantia* L.	*P. stutzeri* (Kx574858)	Bioremediation of Fe- and Mn-contaminated water; enhanced SOD, CAT, proline, carotenoids, and flavonoids; and reduced phytotoxicity and oxidative stress	Improved stress tolerance, increased germination rate, and higher organic acid content in rhizosphere soil	Increased proline, carotenoids, and proline activity in tube-well water in combination with PGPR + NPs	Tariq and Bano (2023)
Wheat	*Azospirillum brasilense*	Synergistic interaction with SCU and Ver, improved tolerance to saline soil, and enhanced proline accumulation and nutrient uptake (N, P, and K)	Increased chlorophyll (a, b, and total), plant height, grain weight, and protein content	Optimized N-use efficiency and reduced environmental stress	Elwan et al. (2025)

#### 3.1.3 PGPR uptake and transport of micronutrients

##### 3.1.3.1 Iron

Globally, the extent of soils affected by micronutrient deficiencies in both pastureland and agricultural systems is increasing. Such micronutrient shortages result from decreased nutrient mobility, availability, and the ability of plants to absorb nutrients from the rhizosphere. The rhizosphere is characterized by high microbial diversity and activity and extends several millimeters into the surrounding soil from the root surface (Kumar et al., 2016). Additionally, the uptake and transport of micronutrients by roots are influenced by soil physicochemical properties, microbial activity, and microbial community composition. In this section, we discuss the major PGPR-mediated micronutrient uptake and transport processes (Table 3) and their impact on plant growth and development.

**Table 3:** Examples of PGPR-mediated micronutrient uptake and transport mechanisms.

Plant	PGPR strain	Mechanism	Effect	Additional observations	References
Groundnut	*B. subtilis*	Enhanced Fe availability (via siderophore production)	Increased pod yield, shoot fresh and dry weight, and Fe availability	Increased Fe, Zn, K, P, and N contents in kernels and straw; ameliorated Fe deficiency	Sarwar et al. (2022)
Potato	*Acinetobacter calcoaceticus* and *B. simplex*	Fe uptake and solubilization; synergistic effect of PGR and tryptophan; edible tissue biofortification	Increased Fe content in tubers and improved nutritional and agronomic traits	Increased soil Fe availability; cost-effective approach	Mushtaq et al. (2024)
Maize, tomato, broccoli, and radish	*Pseudomonas* sp. and *Spirulina platensis*	Functionalization of nanoparticles; interaction of ZnO and *γ*-Fe_2_O_3_ nanoparticles with microbes; enhanced nutrient uptake	Enhanced overall plant growth, shoot diameter, shoot fresh weight, and soil microbial health	Improved Fe assimilation; cost-effective	Guardiola-Márquez et al. (2023)
Rice	ITB (identified by 16S rRNA gene sequencing)	Mobilization of FeSO_4_, FeCl_3,_ and Fe, increased N and P uptake; reduced Fe^2+^ and Fe^3+^toxicity; and phytostimulation under Fe stress	Increased root and shoot growth, N and P uptake, Fe mobilization, and chlorophyll content	Enhanced the soil’s ability to sequester iron and reduced Fe stress	Chandwani et al. (2022)
Barley	*P. fluorescens* (SBW25) and *P. putida* (KT2440)	Uptake of Mn, Co, Ni, Fe, Cu, and Zn; enhanced Ca, S, Si, Mg, K, P, and Al uptake, ACC deaminase; siderophore production; Cl^−^ exclusion	Reduced salinity and drought stress	Improved stress tolerance and uptake of micro- and macronutrients	Zaib et al. (2023)
Wheat	*B. megaterium* CHW-22	Solubilization of Zn (ZnCO_3_, ZnO, and Zn^3^(PO_4_)_2_); production of phytohormones, organic acids, and siderophores	Increased Zn biofortification and nutrient uptake	Significant synergistic effects under pot and field conditions on NUE	Yadav et al. (2023)
*Viburnum grandiflorum*	*A. terreus*	Enhanced plant growth, nutrient absorption, and defense mechanisms; increased Cu, Mn, Ca, Mg, Ni, K, Cr, Fe, and Na uptake	Increased plant height, shoot length, fresh weight, moisture content, proline, root length, and leaf area; potential strain for heavy metal detoxification	Enhanced plant defense, development, and nutrient uptake	Shahzad et al. (2023)
Maize	*Rhizophagus intraradices* and *Micrococcus yunnanensis*	Positive correlation between shoot P content and root colonization, and shoot and root dry weight	Increased shoot Fe, Cu, Na, and Zn concentrations under high boron (B) conditions	Exposure to excess B had a greater mitigating effect in plants colonized by the bacteria and arbuscular mycorrhizae	Abdar et al. (2023)
Wheat	*G. graminis*	Compared with Mn treatment, the Mn reducer on agar enhanced plant growth and Mn uptake from soil. MnSO4 seed treatment increased Mn uptake	Greater reductions were observed in hyphal density than in hyphal length	Increased Mn uptake may contribute to improved plant growth	Marschner et al. (1991)
Wheat	*Azospirillum zeae*	Seed priming with IAA, Zn, and Mn improved wheat growth and yield under dryland conditions	Increased grain yield, grain weight, grains per spike, and tiller number	Increased grain N and P contents and yield	Karimi et al. (2021)

Iron is a micronutrient that is abundantly present in the Earth’s crust, but it is required by plants in trace amounts. In soils, Fe is predominantly present as Fe^3+^ oxyhydroxides, which are poorly soluble and are not easily accessible to plants and microbes. Consequently, Fe^3+^ must be reduced to Fe^2+,^ a process facilitated by plants and microbes. The presence of insoluble Fe compounds in soil limits Fe availability, which disrupts the photosynthetic machinery and adversely affects the growth and productivity of plants such as quince (*Cydonia oblonga*), which is particularly susceptible to Fe deficiency. One study showed that quince plants treated with the PGPR *M. yunnanensis* exhibited greater shoot and root biomass and higher chlorophyll content. Moreover, combined inoculation with *P. fluorescens* and *M. yunnanensis* significantly increased Fe content in quince seedlings. Additionally, treatment with *M. yunnanensis* resulted in higher expression of the Phenylalanine Ammonia-Lyase-1 (PAL1) gene and increased PAL activity. Moreover, this strain increased the levels of phenolic compounds and root citric acid (Rahimi et al., 2020). In calcareous soils, Fe deficiency causes significant yield losses; thus, the PGPR represent a useful strategy for improving Fe nutrition in legumes. Inoculation with *S. fuliginis* increased ferric chelate activity and FRO2 expression, leading to higher Fe content in roots, indicating a positive effect on Fe acquisition. The concentrations of Zn and Mn in trifoliate leaves increased after inoculation with *P. jessenii*, whereas the Zn concentration in roots decreased and FER4 expression increased. Co-inoculation with both strains promoted Fe accumulation in trifoliate leaves, enhanced the expression of the FER4 and IRT1 genes, and improved Fe translocation from the roots to the shoots in soybean (Roriz et al., 2021).

Nanoparticles (NPs) were prepared using the co-precipitation technique with preparations derived from *Spirulina platensis* and *Pseudomonas* spp. Under seedbed conditions, the effects of NPs on seed germination, soil microbial growth, and early plant responses were evaluated. The NPs were functionalized with maghemite (*γ*-Fe2O3; 68 nm) and zinc oxide (ZnO; 77 nm). Following functionalization, the diameters of the nanoparticles increased, ranging from 145 to 233 nm. At concentrations of 75 and 250 ppm, particularly for bacteria-functionalized ZnO- and γ-Fe2O3 nanoparticles, plant height, leaf diameter, and fresh weight of broccoli and radish increased significantly. The nanofertilizers at 75 and 250 ppm also markedly enhanced bacterial growth. These findings indicate that synergistic interactions between the biological components and Zn- and Fe-based nanoparticles in bionanofertilizers positively influence plant development (Guardiola-Márquez et al., 2023). Faba bean is an important food crop cultivated since antiquity, and its seeds contain high protein levels (23–41%) compared with those of other legumes, as well as abundant beneficial secondary bioactive compounds. Recent studies have shown that inoculation with arbuscular mycorrhizal fungi (AMF) and PGPR species, including *B. subtilis*, *B. megaterium*, and *Priestia megaterium* (ZS-3), enhances morphological traits, bioactive compound production, mineral nutrient content, and sustainability (Yilmaz, 2025). Similarly, Zhu et al. demonstrated that combined AMF and PGPR inoculation is an effective strategy for the biofortification of faba bean and for improving sustainable production (Zhu et al., 2025). These microbial functions support sustainable agricultural systems by improving plant mineral nutrition. Moreover, engineering-based microbiome methods represent powerful tools for enhancing plant growth, development, and resilience under changing environmental conditions.

##### 3.1.3.2 Zinc, manganese, and copper

Zn is an essential trace element required for all life forms; however, high concentrations are harmful to biota and negatively impact the microbial community when they accumulate in agricultural soils. Excess Zn uptake by plants disrupts metabolic processes, growth, development, and ultimately reduces crop yield. By enhancing the availability of essential nutrients, stimulating the antioxidant defense system, and reducing Zn uptake, *P. aeruginosa* acts as a bioremediation agent and promotes wheat growth against Zn-induced oxidative stress (Islam et al., 2014). Recent studies have shown that zinc oxide nanoparticles (ZnO-NPs) and PGPR can mitigate heavy metal toxicity in crops, particularly Cd toxicity in wheat, by regulating the antioxidant defense system and TaEIL1 expression, thereby preventing Cd absorption, promoting detoxification, and improving food safety (Sehrish et al., 2024).

The zinc-solubilizing rhizobacteria, such as *Priestia aryabhattai* (CLS2) and *Priestia megaterium* (CLS9), which naturally occur in the canola rhizosphere, facilitate the mobilization of sufficient soluble Zn, thus promoting plant development, yield, and Zn content in canola (Jalal-Ud-Din et al., 2024). The application of PGPR strains, such as *B. amyloliquefaciens* (IUB-34) and *K.* var*iicola* (IUB-96, IUB-80, and IUB-93), in combination with consortia or zinc-coated urea, improved Zn uptake in wheat grains. This was reflected in higher SPAD chlorophyll values, root and shoot lengths, and dry biomass in pot experiments (Anwar et al., 2025). Furthermore, studies have shown that PGPR strains (*P. fluorescens* and *A. chroococcum*) alleviated the phytotoxic effects of biosynthesized ZnO-NPs in eggplants, thereby mitigating nanoparticle toxicity and enhancing plant production under abiotic stress (Solanki et al., 2024).

Manganese (Mn) is an important micronutrient that plays a variety of roles in plant physiology, particularly in electron transport in photosystem II (PSII), chloroplast structure, and N metabolism. It catalyzes a number of enzymatic reactions, including redox reactions, hydrolysis, phosphorylation, and decarboxylation, and it also functions as an activator and cofactor for several metalloenzymes. Additionally, Mn deficiency causes oxidative stress, leading to decreased photosynthetic electron transport, water utilization efficiency, and root activity. Studies have shown that PGPR strains, such as *Bacillus* spp. isolated from the maize rhizosphere (ASH6, ASH19, ASH20, and ASH22) and identified by 16S rRNA gene sequencing, have the ability to solubilize insoluble Mn compounds, enhance the growth of maize seedlings in sand culture, and promote maize growth under Mn-deficient conditions (Ijaz et al., 2021a).

Additionally, insufficient soil fertility and the deterioration of soil health reduce the bioavailability of essential nutrients to plants. Biochar improved the levels of Mn, Cu, K, Ca, Zn, and Ni, as well as plant fresh weight, plant dry weight, root fresh weight, tiller number, and germination rate. The combination of PGPR strains, such as *B. subtilis* and *Paenibacillus azotofixans*, and biochar was effective in improving soil properties, plant growth, and nutritional content in einkorn wheat (Çığ et al., 2021). To increase the bioavailability of micronutrients and macronutrients in calcareous soils, the bioavailability of Mn was enhanced through microbial redox cycling. Furthermore, inoculation with PGPR strains, such as *Leptothrix discophora* and *B. polymyxa*, in combination with mineral fertilizer (NPK), improved wheat yield and soil nutrient bioavailability (Babar et al., 2022). The application of *P. mosselii* (PR5) resulted in increased rice grain yield in pots and significantly enhanced the concentrations of P, Mn, and Zn in the shoots and N, Ca, and Si in the leaves. Additionally, it effectively suppressed naturally occurring blast disease via culture filtrate concentrate (CFC) bacterial foliar application, as confirmed by multivariate analysis (Sultana et al., 2024b).

An optimal Cu content is necessary for proper plant growth and development; it is also an essential trace metal that functions as a cofactor for many enzymes, including ascorbic acid oxidase, superoxide dismutase, and polyphenol oxidase. Conversely, high levels of Cu inhibit plant development and damage vital biological functions. Additionally, stem stunting, root browning, leaf chlorosis, necrosis, irregular root architecture, and structural and ultrastructural abnormalities are visible indicators of metal toxicity stress in plants. The vigor index, plant growth indices, and germination rates of groundnut seeds treated with PGPR in combination with ZnO-NPs were assessed under controlled circumstances (Prasad et al., 1999; Fatnassi et al., 2015). Inoculation with the PGPR strain *Priestia megaterium* (RGKP3) in combination with ZnO-NPs resulted in a maximum germination rate of 92%. This combined treatment greatly increased seed germination, the vigor index, and growth parameters, such as root length, biomass, leaf number, and shoot length, compared with individual applications. In addition, inoculation with *Priestia megaterium* (RGKP3 and RG8) in combination with ZnO-NPs produced higher levels of carotenoids and chlorophyll, which enhanced photosynthetic rates and improved metabolic activity, leading to stress reduction (Fatnassi et al., 2015).

Another study found that alfalfa co-inoculated with PGPR and *Rhizobium* exhibited enhanced plant growth and Cu uptake in metal-contaminated soil, reducing Cu stress and enhancing soil biochemical properties. PGPR strains such as *Paenibacillus mucilaginosus* and *Sinorhizobium meliloti* increased the contents of nutrients (N, P, and K) in plant tissues and enhanced plant growth in Cu-contaminated soil (Ju et al., 2019). Similarly, in a naturally occurring Cu-contaminated soil, two metal-resistant endophytic PGPR strains, *Burkholderia* sp. GL12 and *B. megaterium* JL35, increased the efficiency of Cu phytoextraction in *E. splendens* and *B. napus* (Sun et al., 2016). Analysis of the 16S rDNA gene sequence showed that *Kocuria* sp. (CRB15), isolated from the rhizosphere of *Saccharum spontaneum*, was resistant to Cu, Ni, Cd, Zn, and Pb. Furthermore, inoculation of *B. nigra* with strain CRB15 promoted root and shoot elongation in Cu-treated seedlings (Hansda et al., 2017).

### 3.2 Phytohormones production

**Figure 4:**
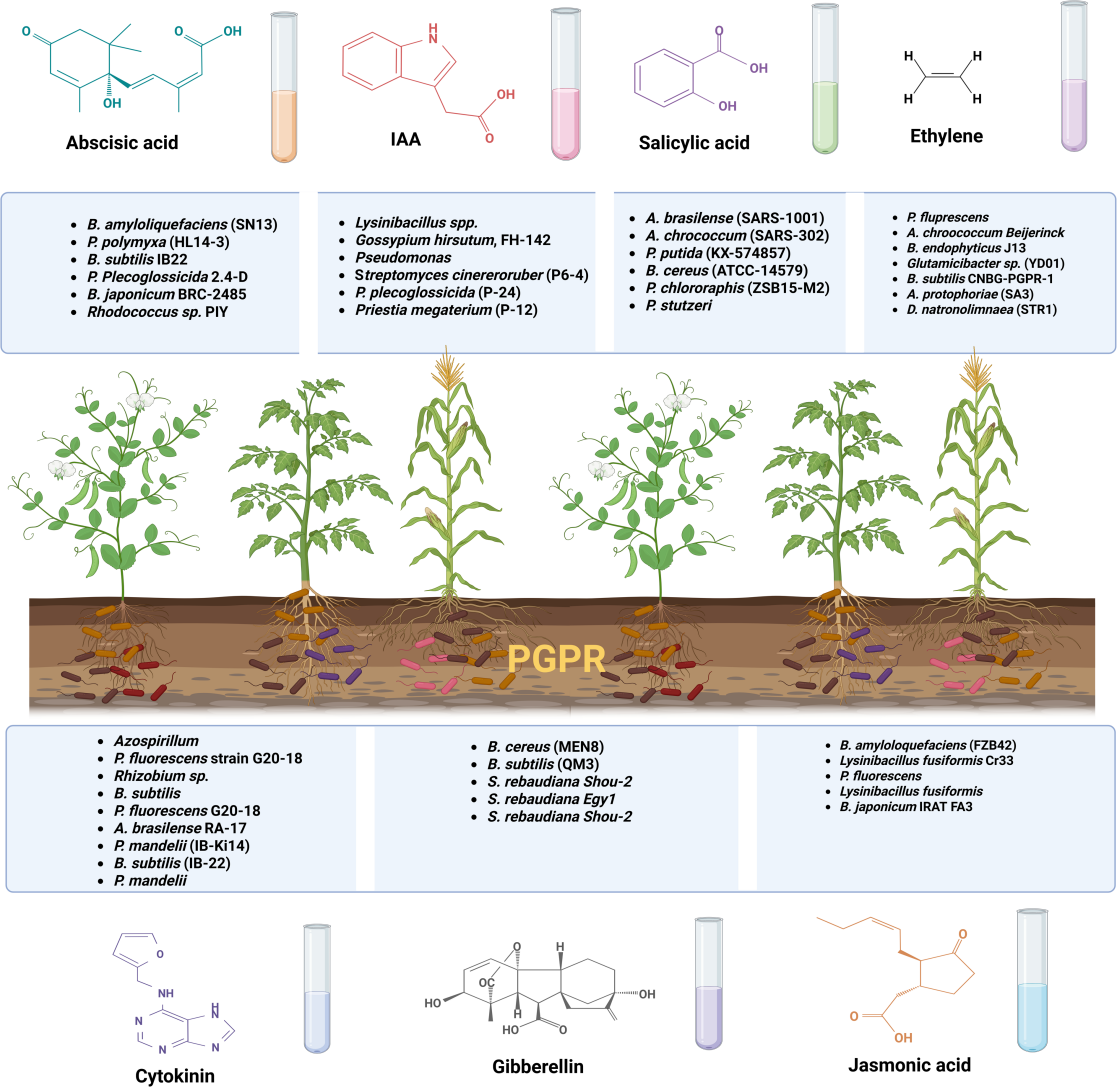
Physiological and functional roles of phytohormones produced by PGPRs: PGPR strains affect plant hormones, including abscisic acid, ethylene, cytokinins, and gibberellins, which have known biological activities. These critical hormones help plants manage stress and stimulate growth, enabling plants to achieve their maximum potential. The aforementioned PGPR strains are examples of microbes that promote tissue resilience, plant growth, and development through hormonal pathways. Created by the biorender.com.

The phytohormones produced by PGPR can regulate growth and development. PGPR-mediated production of these phytohormones influences plant physiological functions, including stress responses, cell division, shoot and root growth, and overall plant growth (Figure 4). A critical unsolved problem is that IAA production alone does not consistently predict growth promotion. Numerous investigations have shown that IAA-positive strains did not improve root biomass under field conditions, perhaps owing to the rapid breakdown of auxin in the rhizosphere or competition from indigenous bacteria. Furthermore, the optimal concentration of bacterially synthesized IAA for growth is species- and cultivar-specific; nonetheless, few studies have determined *in situ* dose–response curves.

#### 3.2.1 Indole-3-acetic acid production

Regarding the microbial potential of PGPR as biofertilizers, PGPR are particularly promising due to their rapid *in vitro* proliferation and the relative ease of large-scale production (Ahemad and Kibret, 2014). Additionally, there is strong evidence regarding the mechanisms underlying PGP, and several studies report increased agricultural productivity following the application of PGPR (Bhattacharyya and Jha, 2012). Previous studies have shown that IAA-producing bacteria are essential for plant growth and development. For instance, the effects of inoculating wheat seedlings with the low-IAA-producing mutant strain *Azospirillum brasilense* (SPM7918) were evaluated in comparison with the wild-type strain Sp6. Compared with the wild-type strain, mutant strain SPM7918 showed a reduced ability to stimulate root system development, highlighting the critical role of bacterial IAA production in root architecture modulation (Barbieri and Galli, 1993; Ramirez and Kloepper, 2012). Auxin is the major naturally occurring phytohormone in plants and regulates a variety of plant growth processes, including root architecture, fruit and embryo development, apical dominance, leaf development, phototropism, leaf abscission, and geotropism. In plants and microorganisms, the most well-understood mechanism for IAA synthesis is the tryptophan-dependent pathway, which plays a central role in auxin-mediated growth regulation (Zhao, 2010).

A recent study identified 24 IAA-producing PGPR strains isolated from the rhizosphere and root tissues of surviving snowbrush plants. In *A. thaliana*, seven isolates (CK-6, CK-22, CK-44, CK-47, CK-50, CK-53, and CK-55) significantly enhanced shoot biomass. Moreover, based on 16S rRNA gene sequencing, except for CK-55, which belongs to the genus *Sphingobium*, all other strains were identified as *Pseudomonas*. All these strains play a key role in promoting plant growth and drought stress tolerance and enhancing the productivity of various cereal and vegetable crops (Ganesh et al., 2024). Another study investigated selected rhizobacterial populations for morphological traits, PGP activities, and biochemical characteristics using 50 selected soil samples from both cultivated and uncultivated plants. Among the isolated strains, M28 produced the highest amount of IAA, tested positive for methyl red and gelatinase, and played a role in N fixation, highlighting its multifunctional PGP potential (Uzma et al., 2025). Bioinformatics analyses revealed that all identified strains of *Lysinibacillus* spp. possessed genes involved in the indole-3-pyruvic acid pathway for IAA biosynthesis, while two strains additionally harbored genes involved in the tryptamine pathway. Inoculation of these strains into *Arabidopsis* (aux1-7/axr4-2) mutant plants altered root architecture; the partial restoration of the mutant phenotype suggested that bacterially produced IAA plays an essential role in regulating plant growth and root development (Pantoja-Guerra et al., 2023). Furthermore, IAA-producing PGPR strains play a key role in plant growth and salt stress tolerance in cotton (*Gossypium hirsutum*, FH-142). These strains were identified through 16S rDNA gene sequencing and exhibited beneficial antimicrobial activity (ST PGPR) within the rhizosphere. In addition, their application enhanced plant growth under saline conditions, indicating their potential as plant growth modulators and biological defense agents in stress-prone environments (Saleem et al., 2021).

Additionally, both tryptophan-dependent and tryptophan-independent pathways are employed by microbes for the biosynthesis of IAA, with L-tryptophan serving as the most effective precursor (Akter et al., 2021). Among bacterial IAA biosynthetic pathways, the indole-3-pyruvic acid (IPyA) route is the most crucial and is widely conserved (Jijón-Moreno et al., 2015). In this pathway, the conversion of IPyA into IAA is catalyzed by an enzyme encoded by the *ipdC* gene, which plays a key regulatory role in the IAA biosynthesis process (Zhang et al., 2021). IAA produced by rhizospheric bacteria influences seed germination, root and shoot development, and total biomass (Babiye, 2022). Recent advances in PGPR research have highlighted their critical functions in maintaining soil fertility, enhancing plant growth, supporting organic agricultural practices, and promoting environmental sustainability (Lata et al., 2024). Consequently, the potential of PGPR has gained scientific attention as an eco-friendly, cost-effective alternative to synthetic fertilizers and pesticides (Baggam et al., 2017). Notably, IAA-producing bacteria from the rhizosphere of chickpeas were morphologically and biochemically identified, with six isolates belonging to *Pseudomonas* and the other two to *Bacillus*. These isolates were able to utilize tryptone as a nitrogen source and sucrose as a carbon source, achieving high IAA levels while also exhibiting phosphate solubilization, indicating their PGP potential. PCR-based molecular confirmation demonstrated the amplification of key genes, including *nifH*, *ipdC*, and *nifK*, which are associated with improved chickpea growth indices (Lata et al., 2024).

Another study showed that bacterial isolates KH14.3 and KH24.1.2, which possess PGPR characteristics, exhibited positive biochemical activity in the IAA assay. Additionally, these isolates exhibited IAA-producing activity and enhanced maize seed germination when applied as seed-coating agents, demonstrating their potential as bioinoculants (Yılmaz et al., 2022). In a related investigation, 16S rRNA gene sequencing identified three PGPR strains isolated from Qassim, Saudi Arabia, namely *Streptomyces cinereroruber* (P6-4), *Pseudomonas plecoglossicida* (P-24), and *Priestia megaterium* (P-12). These strains exhibited multiple PGPR activities, including inorganic P solubilization, IAA production, and siderophore secretion. Their application had a great impact on sustainable agricultural practices, reduced production costs, and enhanced tomato growth in sandy soil (Rehan et al., 2023). Similarly, another study showed that a PGPR *actinobacterial consortium* was able to produce IAA, solubilize phosphorus, and enhance soybean growth under greenhouse conditions. Among the tested actinobacterial strains, *Streptomyces* sp. ASR58 and ASR67, in combination with *Rhizobium* sp., produced the highest IAA concentration compared with each isolate alone and the other bacterial *consortia*. This synergistic interaction enhanced soybean growth (Fatmawati et al., 2023).

#### 3.2.2 Cytokinins

Cytokinins are considered one of the most prominent families of plant hormones, consisting of N6-substituted adenine derivatives, and are essential for cell-cycle progression, among other aspects of plant growth and development (Schaller et al., 2014). According to previous studies, the majority of cytokinins are synthesized within plant tissues; however, certain rhizobacteria also have the ability to produce cytokinins. These bacteria possess cytokinin synthases, which catalyze the transformation of precursor molecules into active cytokinins. It has been discovered that a variety of cytokinins, including cis-zeatin (cZ), isopentenyladenine (iP), and trans-zeatin (tZ), are produced by different rhizobacterial genera, such as *Azospirillum*, *Bacillus*, and *Pseudomonas*. The production of cytokinins (cZ, tZ, and iP) by *P. fluorescens* strain G20-18, as well as the production of tZ and iP by certain *Rhizobium* spp., has been shown to have a beneficial effect on plant growth and development (Taller and Sturtevant, 1999; Spaepen et al., 2007). These cytokinins have been shown to promote seed germination, root formation, and overall plant growth (Kudoyarova et al., 2014). A recent study demonstrated that the phenylalanine metabolism and other important processes associated with plant growth, development, and drought stress tolerance are connected to the genes regulated by *P. fluorescens* G20-18. Cytokinin-biosynthesis-deficient mutants (CNT1 and CNT2) confirmed the capacity of G20-18 to synthesize the plant hormone cytokinin, which plays a role in its interactions with tomato plants. In addition, cytokinin production by G20-18 improves drought stress responses and demonstrates the function of bacterial-derived cytokinins in interkingdom signaling. These findings underscore the potential application of cytokinin-producing rhizobacteria such as G20-18 for improving crop resilience and sustainability in agricultural production systems (Mekureyaw et al., 2022).

The zeatin-type CK-producing *A. brasilense* strains RA-17 and RA-18 were inoculated onto wheat seeds to evaluate their effects on plant growth and yield. Seeds inoculated with RA-18 showed lower growth and yield than those inoculated with RA-17. Moreover, RA-17 enhanced endogenous hormone levels, proline concentration, and enzyme activities in wheat kernels; it also improved grain nutrient uptake, which resulted in increased crop output (Zaheer et al., 2022). Similarly, another study investigated the effects of *P. mandelii* (IB-Ki14), which produces auxin, and *B. subtilis* (IB-22), which produces cytokinin, following inoculation into the rhizosphere of wheat grown in *agrochernozem* soil. In pot experiments, inoculation with either strain enhanced shoot biomass, chlorophyll content, and leaf area in wheat plants. Additionally, IB-Ki14 did not reduce hydraulic conductivity, whereas inoculation with IB-22 increased this parameter. Furthermore, IB-Ki14 facilitated the formation of apoplastic barriers, while IB-22 facilitated cell-wall lignification and decreased the potassium concentration in wheat roots (Akhtyamova et al., 2023). Salt-stressed cultures of *B. subtilis* and *P. mandelii*, which produce auxin and cytokinin, respectively, were applied to the rhizosphere of durum wheat. Inoculation with *P. mandelii* reduced Na^+^ accumulation and enhanced auxin concentration, whereas *B. subtilis* prevented the reduction in potassium and phosphorus concentrations while increasing cytokinin concentration in salt-stressed plants (Martynenko et al., 2022).

#### 3.2.3 Gibberellins

Gibberellins are a class of hormones that play diverse key roles in plants and are primary regulators of fruit and viable seed germination, growth, and reproductive organ development. GAs influence leaf, root meristem, hypocotyl, and stem development by regulating cell division and elongation (Urbanova and Leubner-Metzger, 2016; Plackett and Wilson, 2018). A recent *in vitro* study demonstrated that *B. cereus* (MEN8) was a significant isolate with the ability to increase vegetative growth parameters, such as root and shoot length and fresh and dry weight, in chickpeas. It was also involved in enhancing seed germination, germination energy, germination capacity, germination index, germination percentage, and vigor index. The MEN8 isolate was molecularly identified as *B. cereus* at the species level using 16S rRNA gene sequencing and phylogenetic analysis (Baliyan et al., 2022). Furthermore, a related investigation evaluated growth and physiological indicators in wheat inoculated with *B. subtilis* (QM3). The study found that wheat responded more strongly to GA_3_ treatment than to inoculation with *B. subtilis* QM3 alone. In addition, expression of *TaGA3ox-B2* was not significantly altered by these treatments. According to Pearson’s correlation analysis, a positive correlation was found among *α*-amylase activity, germination rate, and GA content, highlighting the synergistic role of bacterial inoculation and GA signaling in enhancing seed germination (Hu et al., 2024).

The growth and biomass accumulation of Waito-C rice and sesame seedlings were markedly enhanced by nine PGPR isolates. Among these isolates, SIR01, SIR03, and SIR07 were able to produce multiple phytohormones, such as GA-4, GA-9, GA-24, and GA-34, which positively affected the growth of Waito-C rice seedlings. Furthermore, combined inoculation with SIR03 and biochar synergistically increased biomass (fresh and dry weight) and improved stomatal conductance, photosynthetic rate, and chlorophyll fluorescence in Waito-C seedlings (Kang et al., 2022). In another study, *B. subtilis* strains isolated from harsh environments exhibited phytopathogenic potential while possessing PGP abilities. These isolates were characterized by their ability to produce metabolites, form biofilms, produce growth hormones (GA_3_, IAA), sequester nutrients, produce ACC deaminase, and synthesize lignocellulolytic enzymes, which promote the growth and development of *Zea mays* L. (Roy et al., 2024). Additionally, endophytic bacterial strains isolated from the leaves of *Stevia rebaudiana* (Egy1) enhanced plant growth, the production of bioactive compounds, and the expression of genes involved in steviol glycoside (SG) biosynthesis in the *stevia* variety Shou-2. When applied as a biofertilizer in combination with *A. brasilense* and GA_3_, these endophytes increased stevioside content, enhanced SG accumulation, and improved crop yield. Overall, these findings indicate the synergistic role of PGP endophytes and GA supplementation in enhancing *stevia* productivity (Abdelsattar et al., 2024).

#### 3.2.4 Abscisic acid production

Abscisic acid (ABA) is a vital phytohormone involved in numerous plant physiological functions, especially during late seed development. This hormone plays an important role in regulating seed maturation and dormancy and mediating responses to environmental stressors such as cold, salt, and desiccation. Endogenous ABA levels are generally linked with plant growth, as elevated ABA inhibits root elongation and regulates overall vegetative development (Fernando and Schroeder, 2016; Zhang et al., 2022). The regulation of plant hormone homeostasis, particularly ABA levels, has been investigated using the PGPR strain *B. megaterium*. This strain enhanced growth in tomato ABA-biosynthesis mutants (*flacca* and *sitiens*), while it inhibited the growth of ABA-deficient mutant plants. In ABA-deficient plants, *B. megaterium* enhanced ethylene accumulation, which was linked to increased expression of the pathogenesis-related gene Sl-PR1b (Porcel et al., 2014). Similarly, inoculation with the PGPR strain *B. amyloliquefaciens* (SN13) enhanced the development of rice seedlings under drought and salt stress conditions. According to comparative physiobiochemical, nutritional, and antioxidant enzyme analyses, inoculation with SN13 significantly improved rice seedling performance. These effects support its role in modulating interactions between phytohormones and environmental stressors. SN13 also significantly regulated plant nutritional status, metabolic pathways (carbohydrates and fatty acids), and the expression of stress-related genes (Joshi et al., 2023). Recent studies have investigated the effects of inoculation with the PGPR strain *P. polymyxa* (HL14-3) on cucumber RSA, revealing enhanced resistance to oxidative stress and growth inhibition. Additionally, this strain induced ABA-mediated stomatal closure, thereby increasing cucumber resistance to drought stress (Qin et al., 2024).

Additionally, compared with wild-type tomato plants, PGPR improved growth responses, whereas bacterial inoculation hindered the development of ABA-deficient mutant plants, indicating that endogenous ABA production is crucial for plant responses to PGPR. To modulate plant responses to salt stress, the rhizobacterial strain *B. subtilis* IB22 relies on ABA accumulation in the roots of the ABA-deficient barley mutant (Az34). This ABA-mediated response increased leaf hydration, likely as a consequence of increased root hydraulic conductivity, thereby improving salt stress tolerance (Akhtyamova et al., 2021). The quality and biomass yield of vegetable plants cultivated in soils polluted with heavy metals were enhanced by the ABA-producing bacterium *Azospirillum brasilense*, indicating its potential role in stress mitigation under polluted conditions (Wu et al., 2025). A related study in *lettuce* demonstrated that ABA inhibits plant growth under high planting densities. However, the newly identified bacterial strain *P. plecoglossicida* 2.4-D has the ability to degrade ABA, thereby reducing its detrimental effects on lettuce productivity (Vysotskaya et al., 2023). Conversely, *B. japonicum* BRC-2485 can produce ABA, which is reported to be a key factor in activating ISR-related signaling pathways to induce systemic resistance against bacterial wilt disease in tomatoes by induced systemic resistance (ISR)-related signaling pathways (Chattopadhyay et al., 2022). In the forage halophyte *Sulla carnosa*, salt stress reduced dry biomass, whereas inoculation with *B. subtilis* counteracted the effects of salt stress, decreased oxidative stress, and increased expression of the genes encoding SOD and *9-cis-epoxycarotenoid dioxygenase 1* (NCED1), both of which are involved in ABA biosynthesis and stress signaling (Hidri et al., 2024). In addition, when ABA was added as the sole carbon source, the ABA metabolite dehydrovomifoliol was produced, indicating microbial catabolism of ABA. *Rhodococcus* sp. PIY was identified as a rhizosphere bacterial strain that assimilated ABA as its sole carbon source in batch culture, while also modulating ABA levels in plant roots, thereby underscoring the complex function of soil bacteria in ABA turnover and plant hormone homeostasis (Yuzikhin et al., 2021).

#### 3.2.5 Ethylene production

Plant root systems often produce ethylene, a gaseous stress hormone, under water-deficient conditions, which inhibits root development and functions even at extremely low concentrations. Thus, lowering ethylene production in root systems may alleviate the detrimental effects of water stress in plants, allowing them to maintain growth and function under water-deficient conditions (Brunetti et al., 2021). Numerous studies have shown that PGPR strains modulate ethylene-mediated stress. For example, *Pseudomonas fluorescens* and *A. chroococcum Beijerinck*, when used separately or in combination, can influence plant metabolism, antioxidant levels, and photosynthesis, thereby promoting growth under salt stress in the mustard cultivar *Pusa Jagannath*. Among these strains, *P. fluorescens* was the most effective in reducing ethylene stress while simultaneously stimulating photosynthesis and overall plant growth (Khan et al., 2023). Similarly, the PGPR strain *B. endophyticus* J13 enhanced salt stress tolerance in *A. thaliana* by tightly regulating ethylene production and polyamine levels, while also enhancing the brassinosteroid signaling pathway, which collectively improved stress resilience (Nikhil et al., 2024).

Furthermore, a recent study identified the halotolerant PGPR strain *Glutamicibacter* sp. (YD01) through 16S rDNA gene sequencing. When inoculated into rice plants, this strain significantly improved rice tolerance to salt stress by regulating ethylene production, K^+^ acquisition, and ACC oxidase activity (Ji et al., 2020). In addition, the *B. subtilis* strain CNBG-PGPR-1, induced with methionine, was shown to be a crucial regulator of plant tolerance to salt stress by modulating ethylene signaling and ROS scavenging, highlighting its role as a key regulator of stress adaptation (Feng et al., 2024). Finally, abiotic stress tolerance in wheat was enhanced by PGPR strains, including *A. protophormiae* (SA3), *D. natronolimnaea* (STR1), and *B. subtilis* (LDR2). These strains increased IAA content, reduced ABA and ACC concentrations, and modulated the expression of key regulators of the ethylene signaling pathway, including CTR1 and the DREB2 transcription factor, thereby enhancing adaptive stress responses (Barnawal et al., 2017).

#### 3.2.6 Salicylic acid

Salicylic acid (SA) is a key regulator of plant growth and a signaling molecule that plays an important role in stress adaptation and plant development. SA is a primary regulator of solute accumulation and is involved in the regulation of stomatal opening, maintenance of cell turgor pressure, and osmotic water uptake, thereby contributing to plant water relations (Farooq et al., 2010). SA enhances CO_2_ assimilation during photosynthesis and, when applied at optimal concentrations under water-deficient conditions, can improve plant tolerance. Furthermore, plants with adequate endogenous SA levels exhibit better tissue hydration than SA-deficient plants, indicating its protective role against drought stress. Accordingly, adequate endogenous SA levels reduce plant vulnerability to water deficiency and improve drought tolerance. In wheat, foliar application of SA has been reported to improve nutritional quality and enhance the beneficial effects of PGPR on shoot development (Anosheh et al., 2012; El-Bially et al., 2018; Hafez et al., 2019). A recent study showed that the combined application of the PGPR strains *A. brasilense* (SARS-1001) and *Azotobacter chroococcum* (SARS-302), together with SA, reduced the adverse effects of water scarcity, improved nutrient uptake under drought stress, and enhanced wheat productivity (Hafez et al., 2019).

Furthermore, the PGPR strains *B. cereus* (ATCC-14579) and *P. putida* (KX-574857) played an important role in enhancing cold stress tolerance in tomato, although genotypic variation among tomato cultivars also influenced stress tolerance (Tucuch-Haas et al., 2017). In addition, inoculation with the PGPR strain *P. chlororaphis* (ZSB15-M2) in non-autoclaved agricultural soil before rice cultivation improved plant growth, nutrient uptake, root exudate metabolites, and overall plant health through both above-ground foliar and rhizosphere interactions (Bowya and Balachandar, 2020). *Fusarium wilt* and *Ascochyta blight* are the two most significant diseases affecting chickpea; the combined use of SA and *P. stutzeri* significantly suppressed *Fusarium wilt* by enhancing SR in chickpea (Mufti et al., 2023). Similarly, treatment with the PGPR strain *B. cereus*, silver nanoparticles (AgNPs), and SA significantly reduced the severity of yellow rust in wheat, as assessed by the area under the disease progress curve (AUDPC) (Bano, 2020).

#### 3.2.7 Jasmonic acid

Jasmonic acid (JA), its methyl ester (MeJA), and its isoleucine conjugate (JA-Ile) are lipid-derived plant hormones collectively referred to as jasmonates (JAs). MeJA was first identified as a volatile compound emitted by the flowers of *Jasminum grandiflorum*. Initially, JA was recognized as a stress-related hormone and was extracted from the fungus known as *Lasiodiplodia theobromae* as a plant growth inhibitor (Bhatla and Lal, 2023). Inoculation with the PGPR strain *B. japonicum* IRAT FA3 decreased sodium levels in *A. thaliana* under salt stress. This effect was associated with JA priming and altered hydrogen peroxide generation after bacterial inoculation. Moreover, the transcription factors (TFs) MYC2 and JAR1 (JA-conjugating enzyme) were essential for *B. japonicum*-induced shoot development in salt-stressed and non-stressed plants. These findings indicate the key role of JA-mediated signaling in PGPR-induced salt stress tolerance in *A. thaliana* (Gomez et al., 2023). In a related study, JA content, which is directly related to secondary metabolite production and plant defense responses, increased following inoculation with the PGPR strain *B. amyloliquefaciens* (FZB42), thereby promoting root growth of *Glycyrrhiza uralensis* Fisch., a perennial medicinal plant, under drought stress via activation of JA signaling pathways (Yue et al., 2022).

Additionally, inoculation of tomato plants with the root-endophytic bacterial strain *Lysinibacillus fusiformis* Cr33 inhibited Cd absorption by activating JA-mediated signaling pathways, thereby enhancing heavy metal stress tolerance (Zhu et al., 2021).

Furthermore, research has shown that strengthening antioxidant defense mechanisms through increased secondary metabolite synthesis and accumulation enhances the combined effect of methyl jasmonate (MeJA) and the PGPR strain *P. fluorescens,* making mustard plants more resilient to drought stress (Khan et al., 2024). Similarly, the combined application of melatonin and the PGPR strain *Lysinibacillus fusiformis* improved soybean growth through enhanced antioxidant activity, which helped maintain redox homeostasis. Additionally, this combined treatment modulated phytohormone signaling, including a reduction in ABA levels through downregulation of the NCED3 gene, maintenance of stomatal activity to preserve relative plant water status, and increased synthesis of JA and SA. Taken together, these hormonal and redox adjustments contributed to improved drought stress tolerance in soybean plants (Imran et al., 2023).

### 3.3 PGPR role in abiotic stress in plants

**Figure 5:**
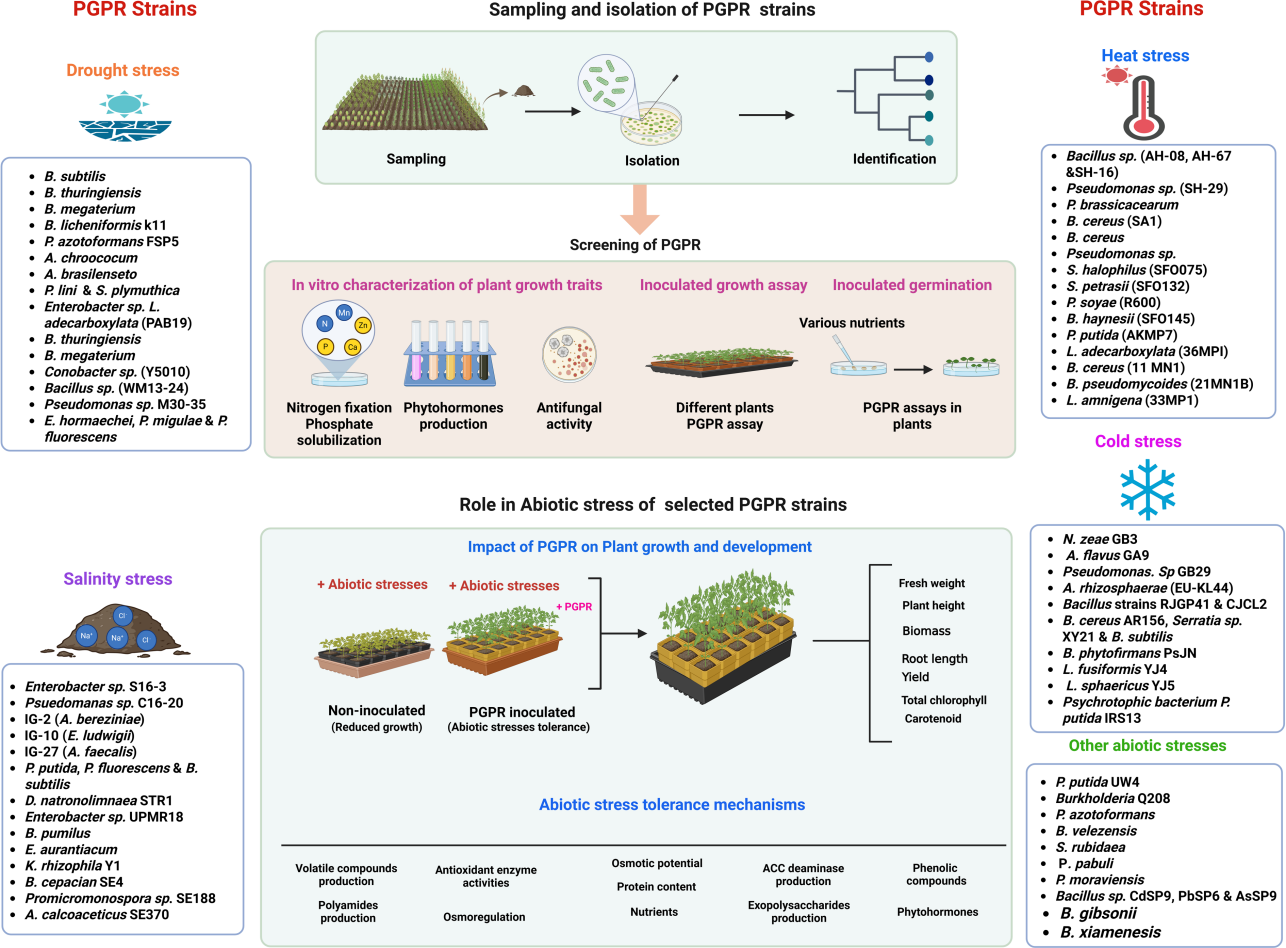
Mechanisms of recently investigated PGPR species, their isolation and screening, and their role in abiotic stresses: This figure categorizes specific PGPR strains that help plants tolerate a variety of abiotic stresses, such as drought, salinity, heat, and cold. The illustrated PGPR strains (e.g., *Bacillus* and *Pseudomonas*) help plants tolerate abiotic stress by producing phytohormones, solubilizing nutrients, and enhancing antioxidant enzyme activity. The figure also illustrates the process of isolating and screening PGPR, together with a summary of their individual roles in enhancing plant growth under stress conditions. Created by the biorender.com.

Plants are severely affected by abiotic stressors, such as extreme temperatures, drought, alkalinity, and salinity, which reduce crop yields and lead to substantial economic losses for farmers. Increasing evidence has shown that PGPR play a key role in mitigating abiotic stress through multiple biological and biochemical activities in the rhizosphere. These beneficial microbes improve nutrient acquisition, enhance oxidative stress responses, strengthen RSA, and regulate osmotic stress responses by modulating phytohormones (Figure 5). A major limitation of the literature summarized in this study is that nearly all studies examine single stresses (drought, salinity, or heat) under controlled conditions. In reality, climate change imposes multiple simultaneous stressors. Only one study (Notununu et al., 2024) addressed combined drought and heat, and none examined triple or quadruple stress combinations. Furthermore, most trials are short-term (<30 days), whereas crop yield losses manifest over full growing seasons.

Plants detect abiotic stress signals through a sophisticated network of downstream TFs, receptors, and secondary messengers that modify gene expression for stress adaptation. PGPR enhance these signaling networks via various mechanisms, e.g., activation of the antioxidant defense system and regulation of stress-responsive TFs (Lephatsi et al., 2021; EL Sabagh et al., 2022). In drought stress conditions, plants synthesize ABA, which is recognized by PYR/PYL/RCAR receptors. These receptors inhibit type 2C protein phosphatases (PP2Cs) and release SnRK2 kinases from negative regulation (Mo et al., 2024; Khan, 2025). Furthermore, the activated SnRK2 kinases phosphorylate downstream AREB/ABF TFs that associate with ABA-responsive elements (ABREs) in the promoter regions of stress-responsive genes, resulting in osmotic adjustment, stomatal closure, and activation of antioxidant genes (Ayub et al., 2025; Zha et al., 2025). PGPR strains such as *B. amyloliquefaciens SN13* (Tiwari et al., 2017), *B. subtilis IB22* (Akhtyamova et al., 2021), and *Paenibacillus polymyxa HL14-3* influence this pathway by either enhancing ABA signaling for stress adaptation or reducing ABA levels to avert excessive growth inhibition (Qin et al., 2024). In addition, abiotic stress triggers excessive production of ROS. At regulated concentrations, ROS serve as secondary messengers that activate stress signaling pathways; however, at elevated levels, they inflict damage on cellular membranes, DNA, and proteins (Zhang et al., 2023). Inoculation with PGPR consistently enhances the expression of antioxidant enzyme genes, including those encoding SOD, APX, GR, and CAT (Gowtham et al., 2022). For instance, inoculation with *P. putida MTCC 5279* (AKMP7) in wheat under heat stress and with the B3P consortium and *Pseudomonas* strains in maize resulted in increased levels of POD, CAT, total chlorophyll, and carotenoids, alongside upregulation of heat-shock protein genes, including HSP1, HSP101, HSP18, and HSP70 (Ahmad et al., 2023; Chauhan et al., 2026). The mitogen-activated protein kinase (MAPK) cascade, which involves sequential phosphorylation through MAPKKK, MAPKK, and MAPK, transmits extracellular stress signals into intracellular responses by phosphorylating TFs such as WRKY and MYB, resulting in the expression of stress-responsive genes (Niekerk et al., 2024). PGPR influence this cascade via phytohormonal regulation. For example, in wheat, the strains *A. protophormiae SA3*, *B. subtilis LDR2*, and *D. natronolimnaea STR1* affect the expression of CTR1 and the DREB2 TF within the ethylene signaling pathway (Barnawal et al., 2017; Giannelli et al., 2023).

Furthermore, under salinity stress, increased Ca^2+^ activates the Salt Overly Sensitive (SOS) pathway, wherein SOS3 interacts with and activates SOS2, which subsequently phosphorylates and activates SOS1, a plasma membrane Na^+^/H^+^ antiporter that extrudes Na^+^ from the cytosol (Kant Nutan et al., 2018; Song et al., 2026), whereas NHX1 sequesters Na^+^ into vacuoles to preserve cytosolic K^+^/Na^+^ homeostasis (Amin et al., 2021). PGPR such as *Beijerinckia fluminensis PVr_9* in *Arabidopsis* upregulate SOS1 and NHX1 genes, and *Dietzia natronolimnaea* in wheat modifies the selectivity of Ca^2+^, Na^+^, and K^+^ to maintain a higher K^+^/Na^+^ ratio under salt stress (Giannelli et al., 2022; Kaleh et al., 2025). Additionally, *B. cereus AR156*, *B. subtilis,* and *Serratia* sp. *XY21* increase the transcription of CBF1, while the psychrotrophic *P. putida IRS13* promotes wheat growth under cold stress through the synthesis of exopolysaccharide (EPS) and IAA, phosphate solubilization, nitrogen fixation, and ACC deaminase activity (Ali et al., 2025b). In addition, under heat stress, heat-shock factors (HSFs) trimerize, translocate to the nucleus, and bind to heat-shock elements (HSEs) in the promoter regions of heat-shock protein (HSP) genes, which serve as molecular chaperones to prevent protein aggregation (Sajjad et al., 2025). The PGPR strain *Bacillus* sp. *SH-16* upregulated HSP15 and HSP30 at 45 °C, whereas SH-16 and AH-67 upregulated HSP100 and HSP65 (Ahmad et al., 2023). Similarly, the B3P consortium enhanced maize growth under heat stress via improved antioxidant enzyme activities (Moradtalab et al., 2020).

#### 3.3.1 Drought stress

Drought is a principal abiotic stress that impedes growth and development, restricts agricultural output, substantially elevates production costs, and ultimately threatens food and feed security. Studies have shown that inoculating plants with PGPR is an effective and eco-friendly strategy that enhances plant development and mitigates the adverse impacts of drought stress (Chieb and Gachomo, 2023). A previous investigation has shown that the combined impact of plant growth regulators (PGRs) (e.g., SA and putrescine) with PGPR strains *B. subtilis*, *B. thuringiensis*, and *B. megaterium* improved drought tolerance in chickpea plants. This integrated treatment enhanced chlorophyll, protein, and sugar concentrations, facilitated osmoregulation, alleviated oxidative stress, and induced the synthesis of stress-responsive proteins. Moreover, the simultaneous application of PGRs and PGPR significantly reduced lipid peroxidation while enhancing leaf area, indicating a synergistic effect that enhanced chickpea growth in sandy soil characterized by moisture and nutrient deficiencies (Khan et al., 2018).

Moreover, pepper plants inoculated with the PGPR strain *B. licheniformis* K11 exhibited enhanced drought tolerance by inducing modifications in protein and gene expression patterns and by producing auxin and ACC deaminase (Lim and Kim, 2013). Similarly, through a variety of metabolic processes, *P. azotoformans* strain FSP5 reduces the adverse effects of drought stress on wheat by regulating multiple metabolic pathways, growth indices, and physiological characteristics under water-deficient conditions (Ansari et al., 2021). Similarly, in another study, the pennyroyal plants (*Mentha pulegium*) were protected by inoculation with PGPR strains *A. chroococum* and *A. brasilense* against water stress. Additionally, these strains significantly reduced damage by improving biochemical and physiological traits and enhancing the biosynthesis of secondary metabolites, which are important for stress adaptation (Asghari et al., 2020). Additionally, inoculation with PGPR strains *P. lini* and *S. plymuthica* significantly lowered MDA and ABA levels, increased antioxidant enzyme activities, and improved soil aggregate stability, leading to increased plant height, shoot and root dry biomass, and relative water content, thereby improving drought tolerance in jujube plants (Zhang et al., 2020).

Moreover, the drought-resistant bacterium *Enterobacter* sp./*L. adecarboxylata* (PAB19) synthesizes substantial quantities of bioactive compounds that promote plant growth and colonizes the roots of mung bean plants under water-deficient conditions (Ahmed et al., 2021). In dry and semi-arid areas, *B. pumilus* has been used as a biofertilizer that reduces drought stress by increasing protein content, osmotic potential, relative water content, and photosynthetic pigments (Yasmin et al., 2017). Under drought conditions, PGRs and PGPR strains (*B. subtilis*, *B. thuringiensis*, and *B. megaterium*) exhibit increased leaf RWC, shoot and root biomass, Fv/Fm ratio, as well as greater accumulation of protein, phenolic compounds, and sugars (Khan et al., 2019). The PGPR strain *Cronobacter* sp. (Y5010) regulates the expression of auxin transporters in drought-stressed maize and efficiently utilizes IAA to promote growth and development (Gao et al., 2023). By controlling the distribution of plant hormones, PGPR strains *Bacillus* sp. (WM13-24) and *Pseudomonas* sp. M30-35 enhance antioxidant enzyme activity, regulate ABA signaling, and sustain plant growth under drought stress conditions (He et al., 2021). PGPR strains, specifically *E. hormaechei*, *P. migulae,* and *P. fluorescens,* were isolated from *foxtail millet* (*Setaria italica L.*), a naturally drought-resistant crop. These strains possess the capability to promote plant growth under drought stress conditions and function as efficient bioinoculants to support agricultural productivity (Niu et al., 2018).

#### 3.3.2 Salinity stress

Salt stress disrupts ion homeostasis (e.g., Na^+^ and K^+^) and impairs mineral uptake in canola plants. Inoculation with *Enterobacter* sp. S16-3 and *Pseudomanas* sp. C16-20 enhanced the K^+^ salinity tolerance index and markedly reduced leaf Na^+^ content in canola plants (Neshat et al., 2022). Similarly, inoculation with PGPR strains IG-2 (*A. bereziniae*), IG-10 (*E. ludwigii*), and IG-27 (*A. faecalis*) enhanced the growth metrics of pea seedlings under salt stress (Sapre et al., 2022). Under saline conditions, PGPR have been shown to increase the essential oil yield of *R. officinalis*, a medicinal plant belonging to the family *Lamiaceae*, highlighting their importance in stress-prone medicinal crop production (Dehghani Bidgoli et al., 2019). In faba beans, inoculation with PGPR strains (*P. putida*, *P. fluorescens,* and *B. subtilis*) improved growth characteristics, including plant height, shoot fresh weight, and leaf area under salt stress (Metwali et al., 2015).

PGPR strains *B. pumilus* and *E. aurantiacum*, inoculated into the salt-tolerant wheat variety Aas-11, together with *P. fluorescens*, applied to the salt-sensitive variety Galaxy-13, are proposed as potential bioinoculants for enhancing wheat growth and yield by modulating morphophysiological and biochemical characteristics under saline conditions (Nawaz et al., 2020). The PGPR strain *Kocuria rhizophila* Y1 alleviates the adverse effects of salinity on maize growth and development under saline stress by modulating plant hormones and nutrient uptake, thereby maintaining ion homeostasis, enhancing photosynthetic efficiency, increasing redox potential, and promoting the expression of the stress-responsive genes (Li et al., 2020). A related study investigated PGPR strains *B. cepacia* SE4, *Promicromonospora* sp. SE188, and *A. calcoaceticus* SE370 for mitigating salt and drought stress in cucumber plants by decreasing the activities of polyphenol oxidase, catalase, and peroxidase (Kang et al., 2014).

#### 3.3.3 Heat stress

Elevated temperatures stimulate the synthesis of several proteins in plants that improve resilience to heat stress. It has been investigated that three *Bacillus* spp. (AH-08, AH-67, and SH-16) and one *Pseudomonas* sp. (SH-29) demonstrated growth and several plant-beneficial characteristics at temperatures up to 45 ± 2 °C. Similarly, under heat stress conditions ranging from 45 to 50 °C, the SH-16 strain showed overexpression of HSPs of 15 and 30 kDa, whereas the SH-16 and AH-67 strains showed upregulation of HSPs of 65 and 100 kDa. Furthermore, inoculation with these strains increased CAT, POD, total chlorophyll, and carotenoid contents while decreasing MDA content, thereby enhancing the overall development of maize during the early stages of growth (Ahmad et al., 2023).

Additionally, under heat stress, inoculation with the PGPR isolate *Pseudomonas brassicacearum* improved antioxidant enzyme activity, while proline and protein contents, as well as the fresh weight of roots and shoots, were markedly increased (Ashraf et al., 2019). Moreover, enhanced physiological fitness under heat stress was demonstrated following inoculation with a consortium of two marine PGPR strains in *Vitis vinifera* cv. *Antao Vaz*. Furthermore, inoculation with these PGPR strains enabled bioaugmented grapevines subjected to heat stress to exhibit improved photoprotection and increased membrane thermostability, resulting in enhanced tolerance to elevated temperatures (Carreiras et al., 2023). Heat stress associated with climate change can negatively affect the growth and yield of temperature-sensitive crops, such as soybean, particularly the cultivar *Pungsannamul*. In this context, inoculation with thermotolerant *B. cereus* (SA1) mitigated heat stress damage in soybean plants by improving physiological performance and stress tolerance mechanisms (Khan et al., 2020).

Moreover, the heat-tolerant PGPR *B. cereus* greatly mitigates heat stress, demonstrating pronounced effects on the morphological and physiological characteristics of tomato cultivars under such conditions. Bacterial inoculation markedly enhanced shoot and root length, leaf surface area, and fresh and dry weight. Under heat stress, increased production of extracellular EPS and the cleavage of ACC into *α*-ketobutyrate and ammonia, mediated by ACC-deaminase-producing bacteria, substantially mitigated the negative effects of heat on tomato development. Another study investigated the combined inoculation of ZnO-NPs and *Pseudomonas* sp. under heat stress in wheat plants, which protected plants against heat and other stresses by increasing proline content, antioxidant enzyme activities (GR, DHAR, peroxidase, catalase, and APX), and ABA levels (Mukhtar et al., 2020). Although focused on salinity rather than heat stress, inoculation with PGPR isolates *P. soyae* (R600), *B. haynesii* (SFO145), *S. halophilus* (SFO075), and *S. petrasii* (SFO132) markedly improved the overall growth of maize under salt stress (Alonazi et al., 2025). Similarly, wheat plants inoculated with *Pseudomonas putida* MTCC 5279 strain (AKMP7) under heat stress showed enhanced root and shoot length, dry biomass, spikelet number, tiller number, and grain development. Moreover, the inoculated plants exhibited reduced heat-stress-induced membrane damage and increased the activities of antioxidant enzymes, including CAT, SOD, and APX, as well as increased cellular metabolite levels, including proline, chlorophyll, proteins, starch, and amino acids (Ali et al., 2011). Similarly, another study showed that inoculation with PGPR isolates *L. adecarboxylata* (36MPI), *B. cereus* (11MN1), *B. pseudomycoides* (21MN1B), and *L. amnigena* (33MP1) mitigated the negative consequences of simultaneous drought and heat stress in maize plants (Notununu et al., 2024).

#### 3.3.4 Cold (chilling) stress

Cold stress is a significant environmental factor limiting crop production because both the intensity and duration of exposure determine the extent of critical damage. Yield losses from cold stress are likely to outweigh those from all other abiotic stresses. PGPR play an important role in agriculture because they can enhance plant development, particularly under stressful (cold/chilling) conditions. Plants have developed unique mechanisms to withstand freezing temperatures and the life-threatening effects of low temperatures (Mishra et al., 2012). In pot experiments, a multi-strain bacterial consortium comprising *N. zeae* GB3, *A. flavus* GA9, and *Pseudomonas* sp. GB29 positively influenced root and shoot length, as well as fresh and dry weight in both garden soil and glacial flour. In addition, these strains promoted wheat growth through siderophore production, phosphate solubilization, zinc production, nitrogen fixation, and regulation of 1-aminocyclopropane-1-carboxylate (ACC) (Irfan et al., 2025). In addition to effectively reducing cold stress, inoculation with the psychrotrophic phosphate-solubilizing isolate *A. rhizosphaerae* (EU-KL44) had beneficial effects on growth, physiological parameters, yield, and nutrient uptake in wheat plants (Kour and Yadav, 2023). Through favorable regulation of ABA, lipid peroxidation, and proline accumulation, the cold-tolerant *Bacillus* strains RJGP41 and CJCL2 positively regulated phytohormone expression, thereby playing a key role in improving wheat growth and development under cold stress (Zubair et al., 2019). Similarly, inoculation of wheat seeds and seedlings with the psychrotrophic PGPR strain IRS14 helped regulate biochemical and metabolic processes, reduced cold stress, and enhanced biomass accumulation and plant growth. It also increased the endogenous levels of phytohormones, antioxidants, proline, carotenoids, and chlorophyll in wheat plants (Ali et al., 2023).

Additionally, treatment with PGPR strains *B. cereus* AR156, *Serratia* sp. XY21, and *B. subtilis* elicited greater and more rapid accumulation of MDA and H_2_O_2_ following the onset of cold stress, thereby triggering mechanisms that reduced cold-induced damage. Furthermore, inoculation with these strains increased the transcription of the stress-responsive gene CBF1 (Subramanian et al., 2016). When tomato plants were exposed to cold stress (15 °C), inoculation with *Arthrobacter* strains OS261, OS211, OB146, and OB155 enhanced seed germination and plant growth in pot experiments. Furthermore, these strains reduced membrane damage, activated antioxidant enzymes, increased proline accumulation, and improved germination under cold stress (Subramanian et al., 2016). Moreover, another PGPR strain, *B. phytofirmans* PsJN, promoted grapevine growth and physiological activity under cold stress by improving root development, biomass, CO_2_ fixation, O_2_ evolution, and the accumulaton of starch, phenolics, and proline (Ait Barka et al., 2006). Under cold stress, inoculation of maize plants with the PGPR strains *L. fusiformis* YJ4 and *L. sphaericus* YJ5 increased the levels of osmolytes, antioxidant enzymes, phenolics, and phytohormones, thereby enhancing resistance to osmotic and oxidative stress induced by low temperatures (Jha and Mohamed, 2023). Similarly, inoculation with the *psychrotrophic bacterium P. putida* IRS13 promoted wheat growth under cold stress through EPS synthesis, IAA production, nitrogen fixation, potassium mobilization, siderophore production, phosphate solubilization, ACC deaminase activity, and its role as a sustainable biostimulant (Ali et al., 2025).

#### 3.3.5 Other abiotic stressors

The main cause of reduced agricultural yields and financial losses in flooded soils is the low oxygen content, which is usually sporadic rather than constant. Flooding fluctuates depending on the weather and season. Although oxygen is normally present in the plant rhizosphere, it is generally maintained at a low level. Researchers are increasingly investigating hypoxic stress because of the potentially detrimental effects of anoxic stress on crop productivity. In cucumber roots, inoculation with the PGPR strain *P. putida* UW4 under hypoxic stress led to changes in gene expression, stimulating the transcription of proteins involved in nitrogen metabolism, carbohydrate metabolism, antioxidant defense, stress responses, binding, and other cellular processes. These proteins cooperate to alleviate hypoxic stress and enhance cucumber growth (Li et al., 2013). At the root surface, inoculation with *Burkholderia* strain Q208 creates a biofilm, inhibits virulence factors and genes involved in survival under hypoxic conditions, and upregulates bd-type cytochrome. Moreover, increased ethylene synthesis promotes aerenchyma formation in roots, thereby facilitating oxygen diffusion throughout the sugarcane cortex (Paungfoo-Lonhienne et al., 2016).

Moreover, microorganisms provide an economical and environmentally friendly alternative for eliminating heavy metals from contaminated agricultural soils. Inoculation with PGPR strains *P. azotoformans*, *B. velezensis*, *S. rubidaea*, and *P. pabuli* reduced the heavy metal stress in pepper by regulating enzymatic and non-enzymatic antioxidants in plant tissues and decreasing the accumulation of heavy metals (Ni, Cd, Pb, and Cu), while increasing photosynthetic pigment content, membrane stability index, and relative water content (El-Saadony et al., 2024). For example, *Pseudomonas* spp. and *Bacillus* spp. have developed resistance mechanisms against certain heavy metals through mobilization, precipitation, immobilization, biosorption, surface complexation, and compartmentalization (Singh et al., 2018). Another study showed that inoculation with PGPR strains *B. cereus* and *P. moraviensis* under heavy metal stress (Pb, Ni, Cu, Cd, Co, and Mn) reduced the harmful effects of these metals and improved the growth and development of wheat plants in sodic soil (Hassan et al., 2017). *Bacillus* spp. strains (CdSP9, PbSP6, and AsSP9) were inoculated into the rice variety *Satabdi* under toxic metal stress. These strains improved the germination rate, relative root elongation, amylase and protease activities, and reduced metal toxicity, thereby improving overall plant growth (Pandey et al., 2013). Similarly, inoculation with the PGPR strains *B. gibsonii* and *B. xiamenesis* improved root and shoot length, fresh weight, antioxidant enzyme activity, and proline content, thereby enhancing the overall growth of *S. sesban* under heavy metal stress in industrially polluted soil (Zainab et al., 2021).

Recent advancements in multi-strain consortia research have shown enhanced efficacy compared with single-strain inoculations under field conditions. One study established the N3P41K9 consortium (*B. firmus MN*3, *B. subtilis PK9*, and *Acinetobacter pittii MP41*) in carrots, resulting in a 12.5% increase in yield, while increasing alkaline-hydrolyzable nitrogen by 72.4%, available phosphorus by 48.2%, and available potassium by 23.7% (Zhu et al., 2024). Similarly, the CONSIII consortium (*S. marcescens ERA6*, *B. proteolyticus ESOB2*, and *E. cloacae ERA9*) improved growth and antioxidant activity in wheat subjected to simultaneous salinity (132 mM NaCl) and heat stress (37 °C) (Staiano et al., 2017). In maize and cotton cultivars, assessment of five PGPR blends (AU8, AU9, TX1, TX2, and TX3) showed that the taxonomically diverse blends TX2 and TX3 markedly enhanced growth in all cultivars, indicating the significance of bacterial diversity in consortium construction (Ayelo et al., 2024). Furthermore, the combination of salt-tolerant microbial strains (*Aspergillus* sp. *SV-1, Bacillus* sp. *RM-1/RM-2*, and *Penicillium* sp. *SV-2*) with organic fertilizer in saline-alkali rice systems resulted in a 218.99% increase in soil organic matter, a 1036.20% increase in available potassium, and a 563.13% increase in available phosphorus, while concurrently enhancing the activities of catalase, sucrase, and urease (Guo et al., 2025). Field-scale studies in potato crops recorded significant yield increases of up to 17%, with Pseudomonas PGPR strains attaining rhizosphere colonization rates of 9.6 × 10^5^ CFU/cm root (Hu et al., 2025). Together, these studies underscore the enhanced effectiveness of various multi-strain consortia under field conditions and the pressing need for standardized validation methodologies.

### 3.4 Role of PGPR in biotic stress

**Figure 6:**
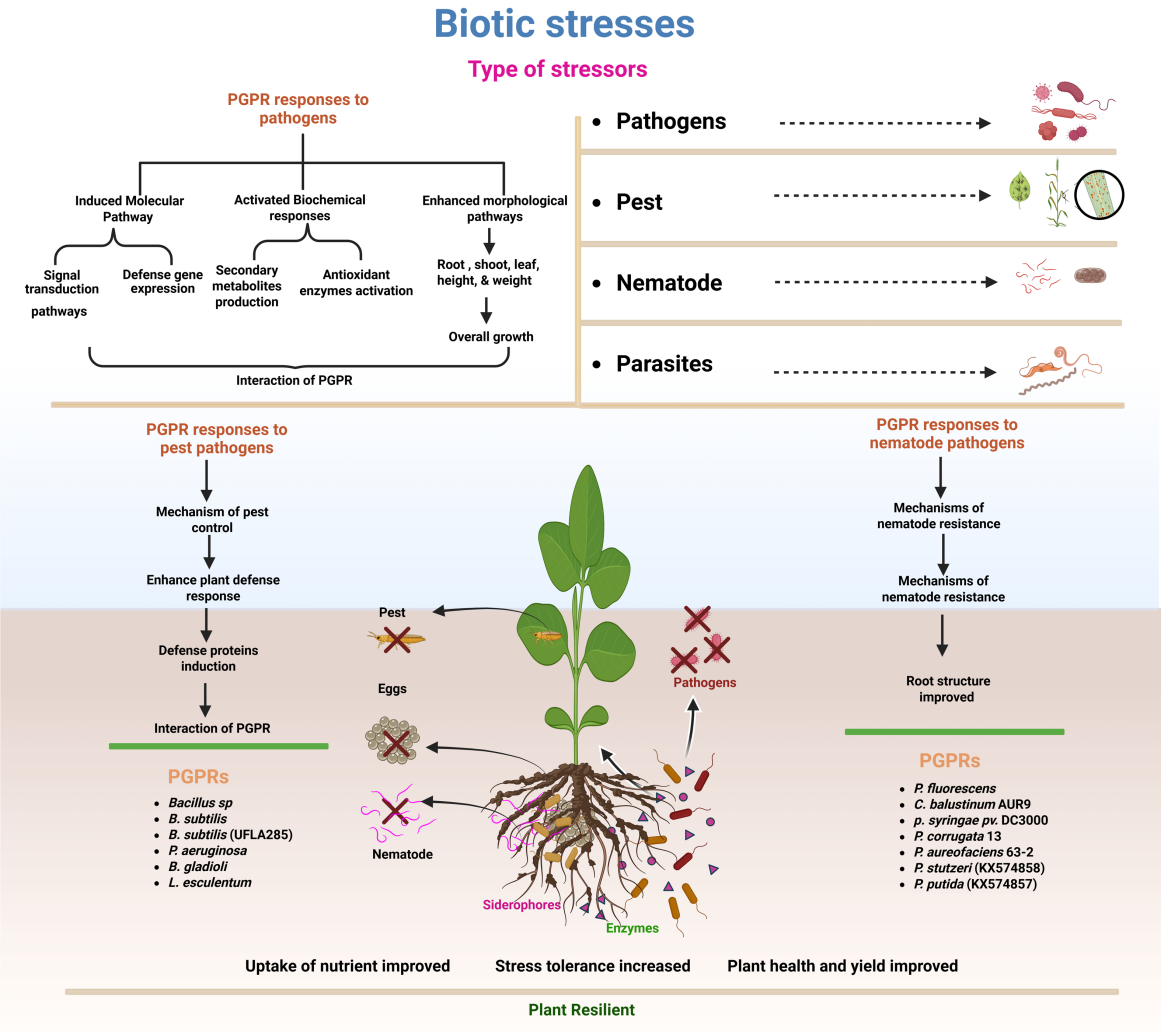
Role of recently investigated PGPR species in biotic stress. This figure summarizes the mechanisms commonly used by PGPR to help plants resist and recover from biotic stresses caused by insect pests, nematodes, and other pathogens. It also illustrates the expression of defense genes and the production of antioxidants. Several specific PGPR strains (e.g., *Bacillus subtilis*, *Pseudomonas fluorescens*) are highlighted, as they serve a dual role in enhancing plant immune responses while improving root development. Created by the biorender.com.

Many rhizobacteria possess growth-promoting properties, some of which are widely distributed and have been commercialized on a large and industrial scale (Figure 6). PGPR have been found to promote the growth and development of a variety of crops (Mahmood et al., 2019a). Among the microorganisms found in the rhizosphere, bacteria are the most prevalent; certain *Bacillus* spp. produce compounds that enhance plant development, protect against pathogen infection, and form long-lived, stress-tolerant spores (Radhakrishnan et al., 2017).

Through the production of numerous secondary metabolites, cell wall-degrading enzymes, hormones, and antioxidants, *Bacillus subtilis* ZE15 strain demonstrates both direct and indirect biocontrol mechanisms to suppress diseases caused by pathogens. Moreover, *B. subtilis* can fix nitrogen, solubilize soil P, and produce siderophores, thereby promoting plant growth while inhibiting pathogens (Hashem et al., 2019). It has been reported that inoculation of cotton plants with *B. subtilis* (UFLA285) resulted in differential expression of transcripts encoding disease-resistance proteins through jasmonate/ethylene signaling and osmotic regulation through the synthesis of proline-related genes (Medeiros et al., 2011).

Additionally, among the many pathogens with a broad host range, root-knot nematodes are a major constraint that causes significant agricultural losses worldwide. Nematode infection reduced seedling growth, caused nuclear damage, increased the accumulation of superoxide anions, H_2_O_2_, and malondialdehyde, and decreased cell viability; these effects were alleviated by microbial inoculation. Inoculation of nematode-infected *L. esculentum* seedlings with the PGPR isolates *P. aeruginosa* and *B. gladioli* reduced oxidative stress, thereby improving overall plant growth and preventing nematode infection (Khanna et al., 2019). Through the production of antibiotics, hydrolytic enzymes (*β*-1,3-glucanase and chitinases), bacteriocins, metabolites such as phytoalexins and siderophores, the PGPR *P. fluorescens* promotes plant growth and induces systemic resistance against diseases, insects, and nematode pests (David et al., 2018). Systemic disease resistance against *P. syringae* pv. tomato DC3000 in *A. thaliana* Col-0 was induced by inoculation with *C. balustinum* AUR9. This response involved activation of the SA-dependent pathway, characterized by increased SA accumulation and enhanced PR1 gene expression, followed by activation of the SA-independent pathway, as indicated by elevated ethylene concentrations (Ramos Solano et al., 2008).

Furthermore, it has been investigated that cucumber roots treated with *P. corrugata* 13 and *P. aureofaciens* 63-28 strains exhibited enhanced PAL activity, thereby alleviating root and crown rot caused by *Pythium aphanidermatum*. Both peroxidase (PO) and polyphenol oxidase (PPO) activities were elevated in the roots as the disease progressed, thereby suppressing *P. aphanidermatum* (Chen et al., 2000). Additionally, to prevent charcoal rot in soybean varieties (Ajmeri and NARC), inoculation with the PGPR strains *P. stutzeri* (KX574858) and *P. putida* (KX574857) increased the activities of polyphenol oxidase, phenylalanine ammonia-lyase, SOD, CAT, and POD, which increased linearly in both Ajmeri and NARC. This treatment in both varieties resulted in noticeably higher accumulations of soluble proteins and leaf proline under infected conditions. It also increased the availability of macronutrients in the rhizosphere of infected soil, and the plant defense and antioxidant enzyme activities were significantly associated with disease suppression (Mufti and Bano, 2019).

### 3.5 Barriers to translating PGPR success from laboratory to open-field systems

The effective transfer of PGPR to open-field conditions is still a major concern, despite decades of study showing the tremendous potential of PGPR under controlled laboratory and greenhouse environments. Only a small number of PGPR-based inoculants have been successfully commercialized and shown to be consistently effective in the field. This disparity between laboratory success and field performance derives from a complex interaction of regulatory and biosafety obstacles, environmental limitations, and formulation challenges that collectively reduce the reliability of PGPR applications in agriculture. The regulatory route to commercialization of PGPR is marked by fragmentation and unpredictability, as every country maintains its own regulatory framework for microbiological commodities, with considerable variation in regulatory requirements among countries. The lack of regulatory harmonization creates serious difficulties for enterprises attempting to commercialize PGPR strains in numerous regions, leading to increased costs and delays in product availability. Biosafety concerns further complicate regulatory approval, as authorities require systematic risk evaluations of the possible effects of introduced microbes on indigenous soil microbiomes, adjacent ecosystems, and human health. The long-term ecological consequences of PGPR adoption are not yet fully understood, resulting in uncertainties that regulators are often reluctant to overlook.

Furthermore, environmental factors represent a significant obstacle to achieving success in the field. The physicochemical properties of soil, such as pH, nutrient status, texture, and organic matter content, exhibit significant variation among agricultural systems and directly affect the survival, proliferation, and bioactivity of PGPR. For instance, a PGPR strain that exhibits remarkable growth in one soil type may completely fail in another owing to variations in pH buffering capacity, the presence of toxic substances, or nutrient availability. In addition to abiotic variables, interactions with indigenous soil microbes significantly influence PGPR efficacy. Recent studies indicate that PGPR substantially enhance plant development in sterilized soils; however, this effect declines markedly in the presence of indigenous microbial populations. Introduced PGPR must compete with indigeneous microbes for nutrients, root colonization sites, and space, often being displaced by more competitive or better-adapted inhabitants. Moreover, the efficacy of PGPR is often assessed under single-stress conditions in controlled settings, whereas field crops generally face multiple concurrent stresses, e.g., drought coupled with heat or salinity combined with nutrient deficiency, that collectively exceed the protective capabilities of individual strains.

Moreover, challenges in the formulation and application further exacerbate these environmental and regulatory obstacles. The absence of efficient formulation methods that preserve microbial inoculant viability during storage, field application, and transportation is considered a key contributing factor to variable effectiveness. Microbial cells must endure desiccation, prolonged storage, and temperature fluctuations while preserving their PGP functions. Although effective inoculants are applied in the field, application methods, including seed coating, foliar spray, and soil drench, must be tailored to each crop and stress condition; however, standardized procedures are largely lacking. Generally, the single-strain liquid inoculants evaluated under laboratory conditions often lack the functional redundancy and synergistic advantages necessary for reliable field performance. Resolving these translational obstacles requires a collaborative and interdisciplinary strategy.

Future studies should emphasize the establishment of standardized field-testing methodologies that account for soil heterogeneity, concurrent stress conditions, and native microbiome competition. Regulatory harmonization across jurisdictions would facilitate commercialization processes, while advancements in formulation technologies, such as protective carriers like biochar and optimized cell protectants such as trehalose, could improve inoculant shelf life and field viability. Collaboration among academic and industry stakeholders is key to bridging laboratory discoveries and agricultural applications, ultimately enabling PGPR to serve as a sustainable solution for climate-resilient agriculture. Recent research on multi-strain consortia has shown encouraging prospects.

## 4 Conclusion and future perspective

Global food insecurity is exacerbated by substantial negative effects on agricultural production. This challenge is further intensified by rapid population growth, biotic and abiotic stresses, ongoing climate change, and a noticeable decline in arable land. Global food security depends on increasing crop production under changing climatic conditions, and PGPR offer a promising approach as biofertilizers, bioremediators, and biopesticides. However, the contradictory results regarding the benefits of PGPR under field conditions require further research and investigation. Additionally, it is necessary to examine the potential contribution of specific strains or consortia to promoting plant development, especially in areas where multiple stressors coexist. So far, this problem has been addressed primarily through molecular methods (such as gene editing), and chemical applications (such as fertilizers and insecticides), rather than through more sustainable farming approaches. PGPR frequently form mutualistic relationships with host plants by enhancing nutrient acquisition through nitrogen fixation, phosphorus and potassium solubilization, and siderophore production. This enhances resistance to biotic and abiotic stresses and regulates plant phytohormones. Similarly, root exudates released into the rhizosphere by host plants serve as a carbon source for phytomicrobiome members, thereby creating a stable environment for the proliferation of microorganisms. Therefore, PGPR application is a readily accessible and underutilized strategy to boost agricultural plant resistance to the various conditions that challenge crop growth, development, and yield. However, environmental factors such as soil temperature, pH, and fertility influence not only plant growth but also PGPR efficiency. This changes the capacity of cultivated plants to generate biomass and food under the environmental stresses linked to climate change.

However, for PGPR technologies to be successfully applied in agriculture, more research is required. The topics that need to be addressed include the formation of consortia, the survival of bacterial strains in the soil, PGPR’s capacity to interact with the soil microbiome, suitable compatibility with the target crop, and adaptation to various environmental conditions. Future research should focus on examining the impact of the microbiome on PGPR interactions and improving plant–PGPR interactions through genetic approaches. Addressing these challenges will require multidisciplinary collaboration among experts in agronomy, microbiology, and genetics. Agro-ecosystems and the rural environment will benefit greatly from green biotechnology in many ways.

The long-term goal of increasing crop output in saline agroecosystems may be attained by understanding the osmoadaptation mechanisms involved in developing SS tolerance, which may help reduce SS and boost the global food supply. In these low-nutrient agroecosystems, increasing the productivity of saline soils will contribute to achieving food security and enhancing the quality and quantity of soil organic matter. Researchers can develop more resilient, effective, and environmentally sustainable agricultural systems that will provide food security while minimizing their adverse environmental consequences through these advancements. This has the potential to help reduce carbon emissions and combat climate change. The reasons for inconsistent findings are not well understood but are likely to arise from variations in soil type, competition among local microbiomes, and the use of single- versus combined-stress experimental methods. The performance of PGPR strains under concurrent drought, heat, and salinity conditions, which increasingly characterize climate-affected agriculture, remains largely unexamined. Until long-term field experiments with multi-stress treatments are standardized, the dependability of PGPR as a reliable biofertilizer will remain ambiguous.
